# Physical and Psychosocial Benefits of Sports Participation Among Children and Adolescents with Chronic Diseases: A Systematic Review

**DOI:** 10.1186/s40798-024-00722-8

**Published:** 2024-05-15

**Authors:** Borja Sañudo, Antonio Jesús Sánchez-Oliver, Jesús Fernández-Gavira, Dominik Gaser, Nicola Stöcker, Miguel Peralta, Adilson Marques, Sofia Papakonstantinou, Chiara Nicolini, Christina Sitzberger

**Affiliations:** 1 Department of Physical Education and Sport, University of Seville, Seville, Spain; 2 Departamento de Motricidad Humana y Rendimiento Deportivo, University of Seville, Seville, Spain; 3 Chair of Preventive Pediatrics, Department of Sport and Health Sciences, Technical University of Munich, Munich, Germany; 4 CIPER, Faculdade de Motricidade Humana, Universidade de Lisboa, Lisbon, Portugal; 5 Faculdade de Medicina, ISAMB, Universidade de Lisboa, Lisbon, Portugal; 6 CRETHIDEV. Creative Thinking Development, Attiki, Greece; 7 CEIPES. Centro Internazionale per la Promozione dell’Educazione e lo Sviluppo, Palermo, Italy; 8 Department of Sport and Health Sciences, Technical University of Munich, Munich, Germany

**Keywords:** Children, Chronic diseases, Psychosocial, Physical fitness, Quality of life, Sports

## Abstract

**Background:**

This study aims to identify sports interventions for children and adolescents (CaA) with chronic diseases and evaluate their impact on physical, psychological, and social well-being. The findings of this study will contribute to our understanding of the potential benefits of sports interventions for CaA with chronic diseases and inform future interventions to promote their overall health and well-being.

**Methods:**

A systematic review was conducted in eight databases. This systematic review followed PRISMA guidelines and utilized a comprehensive search strategy to identify studies on sport-based interventions for CaA with chronic diseases. The review included randomized controlled trials and observational studies that focused on physical and psychosocial outcomes.

**Results:**

We screened 10,123 titles and abstracts, reviewed the full text of 622 records, and included 52 primary studies. A total of 2352 participants were assessed with an average of 45 ± 37 participants per study. Among the included studies involving CaA with chronic diseases with an age range from 3 to 18 years, 30% (n = 15) autism spectrum disorders, 21% (n = 11) cerebral palsy, 19% (n = 10) were attention deficit hyperactivity disorder, and 17% (n = 9) obesity. Other diseases included were cancer (n = 5), asthma (n = 1) and cystic fibrosis (n = 1). Interventions involved various sports and physical activities tailored to each chronic disease. The duration and frequency of interventions varied across studies. Most studies assessed physical outcomes, including motor performance and physical fitness measures. Psychosocial outcomes were also evaluated, focusing on behavioural problems, social competencies, and health-related quality of life.

**Conclusion:**

Overall, sport-based interventions effectively improved physical and psychosocial outcomes in CaA with chronic diseases. Interventions are generally safe, and participants adhere to the prescribed protocols favorably. Despite that, there is little evidence that interventions are being implemented. Future studies should include interventions tailored to meet the common issues experienced by CaA with chronic conditions, providing a comprehensive understanding of the impact of sports interventions on those affected.

***Registration*:**

The methodology for this review was pre-determined and registered in the PROSPERO database (registration number: CRD42023397172).

**Supplementary Information:**

The online version contains supplementary material available at 10.1186/s40798-024-00722-8.

## Key Points

- The study's objective was to examine the impact of sports interventions on CaA with chronic diseases, focusing on physical, psychological, and social well-being.
- The study's findings showed that sport-based interventions effectively improved physical and psychosocial outcomes in CaA with chronic diseases. Moreover, participants generally adhered favorably to the prescribed intervention protocols.
- To enhance the impact of sports interventions, future studies should develop tailored interventions addressing the common issues (e.g., limited accessibility and inclusion, psychosocial barriers, variable adherence, etc.) experienced by CaA with chronic conditions, providing a comprehensive understanding of the benefits of these interventions for affected individuals.

**Supplementary Information:**

The online version contains supplementary material available at 10.1186/s40798-024-00722-8.

## Background

Chronic diseases in children and adolescents (CaA) refer to long-term medical conditions requiring ongoing medical care and management. These conditions can affect a child’s physical, emotional, and social well-being, lasting for months, years, or even a lifetime [1]. Some common examples of chronic illnesses in children include asthma, diabetes, obesity, cystic fibrosis, autoimmune disorders, cancer, and neurological disorders [1]. The prevalence of these disorders varies depending on the specific condition and population studied. About 40% of CaA are affected by at least one chronic disease [2] and 25% had two or more chronic conditions [3]. While the statistics highlight the prevalence of chronic health conditions among CaA, it is crucial to recognize that these conditions can significantly impact various aspects of their well-being, encompassing physical health, psychological resilience, and overall quality of life [4]. Considering the significant impact on a child’s well-being, as well as on their family and caregivers, effective management and support for these conditions are essential to optimize outcomes and quality of life for affected children and their families [5].

Regular physical activity is a significant non-pharmacological approach that can contribute to overall well-being and improve the quality of life [6]. There is evidence showing that effective strategies aimed at optimizing the benefits of physical activity participation can promote health in CaA with a variety of chronic diseases, including obesity [7], asthma [8], cystic fibrosis [9], cancer [10], autism spectrum disorders [11] or attention deficit hyperactivity disorders [12]. However, recent literature show that youth with chronic diseases often do not meet the recommended guidelines and specific considerations in promoting physical activity [13], and while physical activity is considered a cornerstone in the management and treatment of chronic diseases in CaA [14], several studies have identified significant barriers that hinder young individuals with chronic diseases from engaging in these activities [15]. Some of these barriers include physical limitations [16, 17], fear of exacerbating symptoms [16, 17], lack of motivation [16, 17], and lack of opportunities or access [16, 17]. Moreover, CaA with chronic diseases may feel socially isolated or excluded from physical activity and exercise opportunities due to their condition [13, 16, 17]. This situation may result in a diminished sense of social support and a reduced belief in one’s ability to engage in physical activities. Individuals facing chronic diseases may require access to secure and suitable facilities, equipment, or programs to bolster their physical activity aspirations. Consequently, recognizing that obstacles may hinder participation in physical activity, it becomes essential to explore alternative options that can, at the very least, partially mitigate these challenges.

Research suggests that involvement in organized sports tends to remain consistent over time, thereby enhancing the likelihood of maintaining high levels of physical activity into adulthood [18]. Furthermore, participation in sports fosters increased self-confidence, a heightened sense of belonging, an enhanced quality of life, and facilitates opportunities for social interactions [19]. Sport has been shown to improve young people’s physical and psychological function [20]. In CaA with chronic diseases, participating in sports could alleviate the barriers reported for physical activity participation because sports provide opportunities for social interaction, structured and supervised activities, and a sense of belonging to a team or community [21]. Additionally, sports often have clear rules and objectives, which can help individuals overcome the barriers of not knowing what activities to do or how to do them (e.g., by removing uncertainty about what activities to engage in and how to perform them, sports can alleviate the barrier associated with not knowing how to initiate or participate in physical activities) [19, 22]. Regular sports participation can also improve physical fitness, self-esteem, and mental health, which can further motivate individuals with chronic diseases to continue engaging in physical activity [22]. Although there is a well-established understanding that physical activity and sports participation offer significant health benefits for healthy CaA [20, 23], the impact of sports engagement on physical fitness and health-related outcomes among young people with chronic illnesses needs to be more adequately explored [13]. Thus, the focus of this systematic review is to give an overview of (1) which less formal structured physical activities and sports interventions are used in research for CaA with chronic diseases, (2) the impact on physical fitness, physical activity, psychological well-being, social benefits, and overall quality of life of these interventions. Our intention is to establish a clear differentiation between activities that fall under the umbrella of sports or less formal “structured” physical activities and those classified as exercise, which adheres to specific parameters such as frequency, duration, and intensity. With this understanding, our goal is to facilitate the creation of practical applications for engaging in sports participation among various prevalent pediatric chronic diseases.

## Methods

The methodology for this review was pre-determined and registered in the PROSPERO database (registration number: CRD42023397172). This study adhered to the guidelines outlined in the Cochrane Handbook for Systematic Reviews of Interventions and the PRISMA Statement [24].

### Data Sources and Searches

A comprehensive search strategy was developed to identify peer-reviewed journal articles until February 15, 2023. Potentially eligible studies were identified through a systematic search in the following databases: PubMed, MEDLINE, CINAHL, SPORTDiscus, Web of Science, Scopus, PsycINFO and ERIC. This review followed the PICOS framework. The search strategy included the study population, condition and context using terms and keywords derived from preliminary searches and with the assistance of experts in the subject area (see supplementary file 1 for the search strategy). The study population consisted of CaA (< 18 years of age) with chronic diseases (lasting for years or even lifelong), including obesity, asthma, diabetes, haemophilia, cardiovascular diseases (CVD), cancer, cystic fibrosis, epilepsy, developmental disabilities, cerebral palsy, autism spectrum disorders (ASD), attention-deficit/hyperactivity disorder (ADHD) and Post-traumatic diseases. All sport-based interventions were included (intervention). Studies should have either an intervention group with sports and movement-related activities and a control group without targeted sports interventions (e.g., treatment as usual) or only one intervention group (e.g., observational designs); therefore, the primary subset of studies included randomized controlled trials (RCT), but also randomized trials (RT) and observational studies were included. Both physical (e.g., physical fitness, gross motor skills) and psychosocial (e.g., social behaviours, social support, peer relationships, cognitive functions, or health-related quality of life) outcomes were considered. Mental indicators (e.g., self-efficacy, self-esteem, emotional well-being, etc.) were also considered within this group. Limitations on language or publication date were not applied in this study.

### Study Selection

Inclusion criteria were controlled studies including a non-intervention group as a comparison and studies using quantitative comparative observational designs performed in CaA < 18 years old diagnosed with a chronic condition. Studies were excluded if:

(a) not participating in any sports interventions, physical activity or leisure-time activities related to sport, (b) they did not include one of the aforementioned chronic conditions (there was a need for a confirmed medical diagnosis for study eligibility), (c) participants above 18 years old, (d) case reports, non-longitudinal observational studies, qualitative studies, letters, and systematic or narrative reviews, (e) did not include physical or psychosocial outcomes.

In this systematic review, the term “sport” is defined as organized, competitive activities with established rules and involving physical exertion. Less formal “structured” physical activities encompass a broad spectrum of leisure physical activities that may not fall under traditional competitive sports but still involve planned and intentional movement. These activities may include, but are not limited to, recreational games, dance, fitness classes, and outdoor play. To be eligible for inclusion, studies should present interventions involving sports or physical activities.

A two-phase article selection process was conducted. Firstly, the titles and abstracts of the articles were screened, followed by the inclusion phase, where the full text of all articles meeting the inclusion criteria was reviewed. The screening and inclusion phases were carried out independently by two reviewers (BSC and AJSO) who were blinded to each other’s assessments. Any articles that did not meet the eligibility criteria were documented with reasons using an eligibility checklist. Any disagreements between the two reviewers were resolved by a third reviewer (JFG).

### Search and Selection Process

**Fig. 1:**
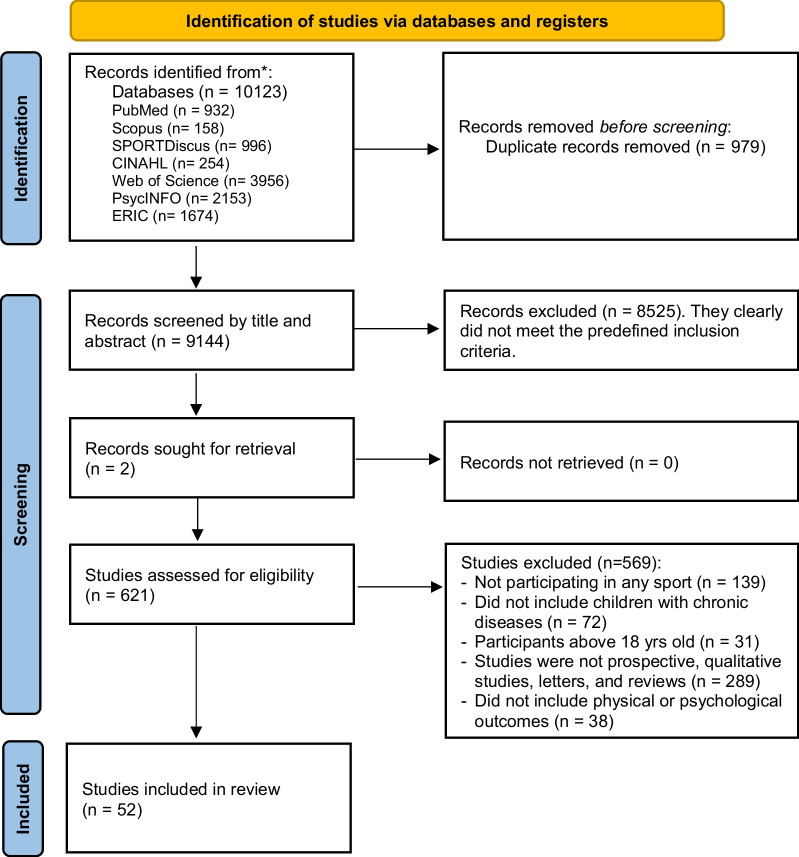
PRISMA flow diagram of literature search and selection process

The flow chart of the selection process is shown in Fig. 1. We identified 10,123 records from the eight databases. From these, 979 duplicates were removed. After the screening of the titles and abstracts, 8525 were excluded. Two sources were acquired externally; consequently, during the inclusion phase, we thoroughly examined the full text of 621 records, assessing the eligibility of all reports. Finally, we identified 52 studies that met the inclusion criteria [25–76].

### Data Extraction

For each study included in the review, data were extracted on general characteristics of the study (author, year of publication, setting -intervention location-, sample size (in controlled studies, the number of participants receiving control and intervention); sociodemographic characteristics (sex, age, chronic disease); description of the intervention (sport, duration, frequency, main results, adverse effects, adherence). Physical and psychosocial outcomes and methods of assessment were also retrieved.

### Methodological Quality

The PEDro (Physiotherapy Evidence Database) scale was used to assess the methodological quality of the studies. It was developed to provide a standardized method for evaluating the internal validity of clinical trials in physiotherapy. The PEDro scale consists of 11 items, each assessing different aspects of the study design, conduct, and analysis. These items include criteria such as random allocation, concealed allocation, baseline comparability, blinding of subjects, therapists, and assessors, intention-to-treat analysis, and statistical reporting. Item 1, related to external validity, was not included in the overall score calculation. The total PEDro score for a study is the sum of scores across the items (from 0 to 10), with a higher score indicating better methodological quality.

## Results

### Description of Studies

A summary of the included studies is reported in Table 1. The studies encompassed a range of designs, sample sizes, and participant characteristics to provide diverse insights into the effects of sport participation on CaA with chronic conditions. All included studies used a longitudinal design; however, 24% did not include a non-intervention comparison group, 18 (34%) were quasi-experiments, and 22 (42%) utilized an RCT design. A total of 2352 participants were assessed, with an average of 45 participants per study (ranging from 6 [71] to 222 [69], per study). The age of the participants ranged on average from (mean ± sd) 4.9 ± 0.6 [32] to 15.0 ± 1.0 [63]. The age range was 3 to 18 years.

**Table 1:** Characteristics of the included studies

							Intervention	
Study	Year	Sample (n or %males); study design	Age (± SD), range	Study population	Setting	Sport/PA	Duration	Duration/Frequency
García-Gómez et al. [25]	2013	16 (EG, n = 8, CG, n = 8). quasi-experimental	range 7–14	ASD	Horse center	Horse-riding	12 wks	60 min, 2 × wk
López-Diaz et al. [26]	2021	15 (100% males)	8.6 (1.1), range 6–12	ASD	Community Sport setting	Soccer	8 months	60 min, 2 × wk
Morales et al. [37]	2021	11 (64% males)	10.2 (2.4), range 9–13	ASD	Judo facility	Judo	8 wks	75 min, 1 × wk
Bo et al. [48]	2019	9 (100% males)	9.2 (1.8), range 8–13	ASD	Community setting	Free play, ball games, Group instruction ball skills	2 wks	210 min, 5 × wk
Lee et al. [59]	2021	19 (% males not reported)	9.3 (3.0), range not reported	ASD	Recreation center	Ball games and dances	8 wks	45 min, 2 × wk
Edwards et al. [70]	2017	30 (EG, n = 11, CG, n = 19) RCT	range 6–10	ASD	Not reported	Kinect Sports Season 2 (Specific mini-games (e.g., baseball, golf, tennis, table tennis, soccer, bowling, volleyball, and football)	2 wks	45–60 min, 3 × wk
Wang et al. [73]	2020	59 (EG, n = 30, CG, n = 29) quasi-experimental	5.1 (0.6), range 3–6	ASD	School	Mini-basketball	12 wks	40 min, 5 × wk
Pan et al. [74]	2010	16 (EG, n = 8, CG, n = 8) quasi-experimental	7.2 (1.2), range 6–9	ASD	Local indoor hydrotherapy and swimming pool	Swimming	10 wks	90 min, 2 × wk
Guest et al. [75]	2017	13 (0% males)	9.7 (1.0), range 8–11	ASD	Summer camp	Track and field, basketball, soccer, and baseball	1 wk	Not reported
Tse et al. [76]	2019	40 (EG, n = 19, CG, n = 21) RCT	9.9 (1.1), range	ASD	School gymnasium	Basketball	12 wks	45 min, 2 × wk
Fragala-Pinkham et al. [27]	2011	12 (EG, n = 7, CG, n = 5) quasi-experimental	9.6 (2.6), range 6–12	ASD	YMCA	Swimming	14 wks	40 min, 2 × wk
Rafiei Milajerdi et al. [28]	2021	60 (SPARK: 20; Kinect: 20; CG: 20). (95% males) RCT	8.2 (1.5), range: 6–10	ASD	Not reported	Sports, Play and Active Recreation for Kids (SPARK); exergaming Tennis (Kinect)	8 wks	24 sesions (14-h intervention: 35 min), 3 × wk
Hassani et al. [29]	2022	30 (66.7% male) (ICPL: 11; SPARK: 10; CG: 9) RCT	8.8 (0.8), range: 8–11 years	ASD	Indoor sessions	I Can have a physical literacy (ICPL); Sport, Play, and Active Recreation for Kids (SPARK)	8 wks	16 sessions (60 min): 2 × wk
Pan et al. [31]	2017	22 (EG, n = 11; CG, n = 11) RCT	9.1 (1.7), range: 6–12 years	ASD	Multipurpose room at the university	Table tennis	12 wks	24 sessions: 70 min, 2 × wk
Cai et al. [32]	2020	29 (EG = 15, CG = 14) (86,2% males) quasi-experimental	4.9 (0.6) range: 3–6 years	ASD	Basketball Pitch	Mini-Basketball	12 wks	40 min, 5 × wk
Gercek et al. [33]	2022	19 (EG, n = 9, CG, n = 10) quasi-experimental	8.3 (2.1), range 6–12	Cerebral palsy	Golf clubs	Virtual and traditional golf training	12 wks	60 min, 3 × wk
Chiu et al. [77]	2014	62 (EG, n = 32, CG, n = 28) RCT	9.5 (1.9), range 6–13	Cerebral palsy	Home	Home-based Wii Sports Resort training	12 wks	40 min, 3 × wk
Hilderley et al. [35]	2022	20 (EG, n = 11, CG, n = 9) RCT	12.0 (2.6), range 8–17	Cerebral palsy	Therapy rooms or gymnasiums	Movement skills (e.g., run, jump, and kick) applied in a sports or athletics	6 wks	45 min, 2–3 × wk
Clutterbuck et al. [36]	2021	54 (EG, n = 29, CG, n = 25) RCT	8.9 (2.0), range 6–12	Cerebral palsy	Community therapy centre	Soccer, netball, T-ball and cricket	8 wks	8 sessions: 60 min, 1 × wk
Ross et al. [38]	2017	97 (51% males)	11.4 (3.1), range 6–18	Cerebral Palsy	Local community center	Swimming, tennis, dance, martial arts, basketball, soccer, baseball, and adaptive cycling	6 wks	360 min, 5 × wk
Pourazar et al. [39]	2018	30 (100% males) (EG, n = 15; CG, n = 15) RCT	11.2(0.8), range: 7–12 years	Cerebral palsy	Virtual Reality	Virtual Reality Games: Bowling and golf	4 wks	25 min, 3 × wk
Clutterbuck et al. [40]	2022	54 (EG, n = 29; CG, n = 25) RCT	8.8 (2.0), range: 6–12 years	Cerebral palsy	Not reported	Sports-specific gross motor activity training, sports education, teamwork development and confidence building for four sport: soccer, netball, T-ball and cricket	8 wks	8 sesions (8 h: 60 min, 1 × wk)
Zoccolillo et al. [41]	2015	22 (EG, n = 11; CG, n = 11) cross-over RCT	6.9 (1.9), range: 4–14 years	Cerebral palsy	Outpatients clinic	Virtual Reality Games	8 wks	16 sessions (30 min): 2 × wk
Polat et al. [42]	2020	44 (11 girls, and 33 boys) (EG = 22; CG = 22) quasi-experimental	7.8 (2.5), range: 4–11 years	Cerebral palsy	At home	Sport activity movements including basic gymnastic positions	8 wks	40 sessions: 50 min, 5 × wk
Feitosa et al. [43]	2017	17 (70.6% male)	10.6 (1.7), range: 7–14 year	Cerebral palsy	Not reported	Adapted Sport: Swimming and seven a side soccer (soccer, n = 11, swimming n = 4, soccer and swimming n = 2)	1 year	Soccer (52 sessions); swimming (104 sessions) both (156 sessions)
Lai et al. [44]	2022	58 (43% males) EG, n = 29; CG, n = 29) RCT	14.0 (3.0) range: 8–17 years	Cerebral palsy	At home (asynchronous training)	Music video movement	4 wks	12 sessions, 3 × wk
Verret et al. [45]	2012	21 (EG, n = 10; CG, n = 11) quasi-experimental	9.1 (1.1), range: 7–12	ADHD	School gymnasium	Basketball, soccer, exercise stations, and tag and ball games	10 wks	45 min, 3 × wk
Pan et al. [30]	2016	32 (EG, n = 16, CG, n = 16) quasi-experimental	8.9 (1.5), range 6–12	ADHD	University (table tennis center)	Racket sport (table tennis)	12 wks	70 min, 2 × wk
López-Williams et al. [46]	2005	63 (92% males)	9.1 (1.7), range 6–12	ADHD	The summer treatment program	Sports skills training (basketball, soccer, baseball, and swimming)	8 wks	8 h/day, 5 × wk
O´Connor et al. [47]	2014	98 (EG, n = 52, CG, n = 46) quasi-experimental	6,6 (0,6), range 5–8	ADHD	Sports centre	Soccer and tee ball	8 wks	9 h, ≈3 h x day
Pan et al. [49]	2017	24 (EG, n = 12, CG, n = 12) quasi-experimental	9.6 (2.5), range 7–14	ADHD	Gymnasium at the university	Horse-riding	12 wks	90 min, 1 × wk
Ziereis and Jansen [50]	2015	43 (EG1, n = 13, EG2, n = 14, CG, n = 16) quasi-experimental	9.4 (1.4), range 7–12	ADHD	Gymnasium at the university	Sport games (e.g., Beach volleyball, handball, throwing and catching) climbing, wrestling games, gymnastics, track and field, sprint and hurdling	12 wks	60 min, 1 × wk
Hupp and Reitman [51]	1999	10 (EG, n = 3)	8.7 (1.1), range 6–10	ADHD	Elementary school campus	Basketball	3 wks	210 min, 5 × wk
Månsson et al. [52]	2019	128 (EG = 64; CG n = 64) (85.16% male) quasi-experimental	11.5 (1.3), range: 10–14 years	ADHD	Local shooting associations	Target-shooting sport	24 wks	24 sessions: 20–45 min, 1 × wk
Benzing and Schmidt [53]	2019	51 (EG = 28; CG = 23) (82.4% male) RCT	10.6 (1.3), range: 8–12 years	ADHD	At home	Exergaming—Xbox Kinect (Microsoft, Redmond, WA)	8 wks	24 sessions: 30 min, 3 × wk
Kadri et al. [54]	2019	38 (EG = 20; CG = 20) (95% male) RCT	14.3 (3.2), range:	ADHD	Private martial arts facility	Taekwondo	1.5 years	50 min, 2 × wk
Walker et al. [55]	2003	95 (EG; n = 57, CG, n = 38) quasi-experimental	13.1 (3.4), range 9–18	Obesity	Summer Camp	Skill-enhancing physical activity sessions	4 wks	60 min, 6 sessions
Cristian-Cosmin et al. [56]	2022	28 (EG, n = 14, CG, n = 14) quasi-experimental	9.4 (1.0), range 8–11	Obesity	School gymnasium	Volleyball	24 wks	90 min, 3 × wk
Cvetković et al. [57]	2018	42 (EG, n = 14, EG2, n = 14, CG, n = 14) RCT	Range 11–13	Obesity	Outdoors on artificial grass	Football	12 wks	60 min, 3 × wk
Cliff et al. [58]	2007	13 (36% males)	10.4 (1.2), range 8–12	Obesity	School	Six locomotor skills (run, gallop, hop, leap, horizontal jump, slide) and six object-control skills (two-handed t-ball strike, stationary dribble, catch, kick, overhand throw, underhand roll) in a fun and enjoyable context	10 wks	120 min, 1 × wk
Griffin et al. [60]	2013	43 (39% males)	10.4 (0.6), range 8–12	Obesity	University gymnasium	Swimming + physical education (e.g., balloon volleying with short-handled rackets, passing, dribbling, and trapping with a partner)	3 wks	45 min + 60 min, 5 × wk
Jette et al. [61]	1977	21 (100% males) (EG, n = 11; CG, n = 10). quasi-experimental	15.3 Years	Obesity	High school facility	Lacrosse	20 wks	45 min aprox., 2 × wk
Korsten-Reck et al. [62]	1994	62 (56.5 males)	10.3 (1.6) range 9–12 years	Obesity	Swimming Pool, Gymnastic hall	Swimming, gametype activities, rhythmic activities and endurance walking are included	24 wks	60 min, 3 × wk
Lofrano-Prado et al. [63]	2022	72 (34 boys), (EG, n = 37; CG, n = 37) RCT	15.0 (1.0), range 13–18 Years	Obesity	Clinical setting	Team sports, circuit training, active games, and physical challenges	12 wks	60 min, 2 × wk
Seabra et al. [64]	2016	88 (100%), EG, n = 29; CG1, n = 29; CG2, n = 30 quasi-experimental	10.3 (1.3) range 8–12 Years	Obesity	Local soccer club	Football	24 wks	60–90 min, 3 × wk
Speyer et al. [65]	2011	30 (60% males) Cross-over RT	13.6 (2.9), range 9–18	Cancer	Hospital facility	Ball games (Soccer, handball, volleyball), racket sports (Tennis, badminton, squash), fighting activities (English boxing, French boxing, fencing, karate), etc	< 4 wks	30 min, 3 × wk
Saultier et al. [66]	2021	80 (EG, n = 41, CG, n = 39) RCT	10.4 (0.5), range 5–18	Cancer	In-hospital and outdoor activities	Multi-activity sessions (dance, basketball, badminton, yoga, skiing, swimming, paddling, etc.)	3 wks + two weekend + long stay 5 days	90–240 min, 5 × wk
Hamari et al. [67]	2019	36 (EG = 17; CG = 19) (72.2% male) RCT	7.8, range: 3–16 year	Cancer	Both during hospitalization and at home	Active video games—Nintendo WiiFit™ games	8 wks	56 sessions: 30 min, 5 × wk
Howell et al. [68]	2018	78 (EG = 53, CG = 25) (44.9% males) RCT	12.7 (1.1), range: 11–15 years	Cancer	At home	Interactive website designed to encourage physical activity via rewards	24 wks	Voluntary
Li et al. [69]	2018	222 (EG = 117; CG = 105) RCT	12.6 (2.0); range: 9–16	Cancer	Campsite	Climbing, trampoline, Mini Olympics	4 days	4 sessions
Westergren et al. [71]	2016	6 (67% males)	10.5 (0.5), range 10–12	Asthma	School gymnasium	Active play (e.g., ball and team games and games of ‘tag’	6 wks	60 min, 2 × wk
Hakim et al. [72]	2022	70 (EG, n = 35, CG, n = 35) RCT	10.1 (1.4), range 8–12	Cystic Fibrosis	Not reported	cycling, swimming, walking, dancing, playing ball, skipping ropes, jumping, upper extremity stretching and gymnastics	1 wk	30–45 min, 4 sessions

*ADHD* Attention-Deficit/Hyperactivity Disorder, *ASD* Autism Spectrum Disorder, *EG* Experimental group, *CG* Control group, *RCT* Randomized controlled trial. *YMCA* Young Men´s Christian Association

The included studies were categorized based on the type of chronic diseases, considering the following categories: ASD (n = 15), cerebral palsy (n = 11), ADHD (n = 10), obesity (n = 9), cancer (n = 5), asthma (n = 1) and cystic Fibrosis (n = 1).

The systematic review included a diverse range of interventions that interventions took place in various settings and facilities. These included schools, high schools or universities’ gymnasiums, summer camps, sports centres, community facilities (e.g., shooting associations), homes (e.g., active video games), golf clubs, community therapy centres, clinical settings (e.g., outpatient clinics), horse centres, campsites, judo facilities, local indoor hydrotherapy and swimming pools, YMCA facilities, basketball pitches, gymnastic halls and local soccer clubs. The duration and frequency of the interventions varied across the studies. Some studies implemented interventions that lasted for one week [69, 72, 75], while others extended over eight months [26], one year [43] or even one and a half years [54]. The prescribed exercise frequency in the selected studies also differed, with some studies offering sessions multiple times per week (e.g., five sessions per week), while others provided interventions on a less frequent basis (e.g., one session per week), with most of the studies offering 2–3 sessions per week [25–35, 39, 41, 44, 45, 47, 53, 55–63, 65–68, 70–72, 74–76].Further details can be found in Table 1.

Physical and psychosocial outcomes are reported in Table 2. Physical outcomes were included in 41 studies, while psychological outcomes were reported in 41 out of 52 studies. Motor performance (e.g., gross motor skills) was assessed in 35 studies [25–35, 37–50, 52, 53, 59, 65–67, 70, 73–76]. Physical fitness was evaluated in 21 (50%) studies. The static and dynamic balance were evaluated in nine studies [29, 32, 33, 38, 42, 49, 50, 56, 66], including measures such as the time standing on the left and right foot and the flamingo balance test.

**Table 2:** Physical and psychosocial outcomes, adverse events and adherence to the interventions included in the systematic review

Study	PEDro scale (from 0 to 10)	Physical Outcomes	Psychosocial Outcomes	Results	Adverse effects	Adherence
*Studies on ASD*						
García-Gómez et al. [25]	2		Adaptive skills, social skills, leadership, withdrawal, anxiety, depression, behavioural problems, atypicality, aggressiveness, hyperactivity, attention problems, and somatization. HRQoL	Significant improvements in aggressiveness, "Interpersonal relations" and "Social inclusion"	Not reported	Not reported
López-Diaz et al. [26]	1	Motor skills	Social Skills	Improvement pre-post in both motor skills and social skills	Not reported	Not reported
Morales et al. [37]	3		Autism-related behaviors and social communication difficulties	Significant main effects in four of the six subscales: repetitive behaviours, social interaction, social communication and emotional responses	Not reported	Not reported
Bo et al. [48]	3	Gross motor skills on locomotor and ball skills	Social communication skills and behaviors	Significant main effects (pre-post) on locomotor, ball skills, and gross motor skills	Not reported	Not reported
Lee et al. [59]	2	Gross motor skills	Social Skills	Significant improvements in object-control skills for the participants. Modest improvements in their performance of the target social skills	Not reported	Not reported
Edwards et al. [70]	2	Gross motor skills	Perceived competence	No significant between-group improvement in actual skill or skill perception. Participants improved (pre-post) their perceptions of skill	Not reported	Not reported
Wang et al. [73]	4		Executive functions, Social Communication Impairment, Repetitive behaviors	Significant improvement on working memory, on inhibition, on regulation, social communication and repetitive behavior	Not reported	Not reported
Pan et al. [74]	8	Aquatic skill measures	Social behavior	Significant social improvements were seen together with improvements (pre-post) in the aquatic skills in four out of five stages measured. No significant differences between groups	Not reported	Not reported
Guest et al. [75]	2	Gross motor skills	Physical self-perceptions, Social and adaptive behaviour	Motor skills significantly improved (pre-post), accompanied by enhancements in physical self-perceptions, self-efficacy, and social skills. However, there were no significant changes in other subscales of physical self-perception	Not reported	Not reported
Tse et al. [76]	6		Social responsiveness and social communication skills; response inhibition and impulsivity; Executive functions (inhibition control and working memory)	Significant changes (pre-post) in inhibition control and working memory but no significant between-group changes were observed	Not reported	97.8%
Fragala-Pinkham et al. [27]	4	Swimming skills, Muscle endurance, Mobility skills	Satisfaction	No significant between-group differences were found. Improvements (pre-post) in swimming skills were observed	No adverse events reported	79–100%
Rafiei Milajerdi et al. [28]	6	Motor skills, balance	Executive functions	Significant (pre-post) changes for aiming and catching in the SPARK (physical activity) group. No significant changes in manual dexterity, balance or executive functions	Not reported	Not reported
Hassani et al. [29]	5	Motor skills; Running speed and agility, balance, bilateral coordination, and strength		Significant between groups changes in motor skills. Significant improvements (pre-post) in running and speed agility, balance, bilateral coordination, and strength	Not reported	Not reported
Pan et al. [74]	4	Motor skills	Executive function; social behavioural measures	Improvements in motor skill proficiency and executive function	Not reported	88%–90% No dropout
Pan et al. [31]	4	Motor skills	Executive function	Main effect (pre-post) on three motor-area composites (i.e. manual coordination; body coordination; strength and agility) and executive function	Not reported	88%-90%
Cai et al. [32]	6	Physical fitness (Running speed and agility, balance, flexibility, and strength)	Social Responsiveness	Improvements (within and between) in running speed, strength and Social Responsiveness	Not reported	Not reported
*Studies on cerebral palsy*						
Gercek et al. [33]	3	Aerobic capacity, flexibility, muscular endurance, balance, spasticity level, gross motor skills		Decreased gastrocnemius and soleus spasticity. Increase (pre-post) in sit-and-reach, lateral step-up, six min walk, and curl up test scores. Between Groups only in balance	Not reported	Not reported
Chiu et al. [77]	9	Coordination, strength, or hand function		Wii™ training did not improve coordination, strength, or hand function	No serious adverse events	96%
Hilderley et al. [35]	8	Gross motor skills, aerobic capacity, lower limb strength,	Self-efficacy, goal achievement, child perceptions of goal performance and satisfaction	Significant improvements in perceptions of goal performance and satisfaction. Measures of goal achievement or fitness did not differ between groups	No adverse event occurred	90%
Clutterbuck et al. [36]	6	Gross Motor Function, Muscle Power Sprint Test, Sprint Test, Vertical Jump, Broad Jump, and Seated Throw		Significant between groups improvements in all fitness measures but the seated throw	Modifications to activities were reported	> 75%
Ross et al. [38]	2	Mobility, cardiorespiratory fitness, walking speed, gross motor skills		Significant improvements in the Timed Up and Go, modified 6-min walk distance. There was no significant change in the overall means for the 25-ft walk/run	Not reported	Not reported
Pourazar et al. [39]	6	Motor skills (reaction time)	HRQoL	No significant between group differences were found. Reaction time measures significantly improved (pre-post)	Not reported	Not reported
Clutterbuck et al. [40]	2	Physical competence; walking; running; jumping; throwing; sports participation	HRQoL	Improvements in sports participation and activity goals and sports-specific physical competence	Not reported	Not reported
Zoccolillo et al. [41]	4	Motor skills		Significant (pre-post) improvements in the motor upper limb abilities in EG. Manual abilities for performing activities of daily living benefited more from conventional therapy	Not reported	Not reported
Polat et al. [42]	6	Gross Motor skills, Walk, Balance	Impact on Family	No significant time or between group differences were found	Not reported	Not reported
Feitosa et al. [43]	4	Mobility and physical function	HRQoL, biopsychosocial profile	Significant (pre-post) improvement in mobility, upper extremity function and global function. The biopsychosocial profile was also improved	Not reported	Not reported
Lai et al. [44]	4		Enjoyment, HRQoL	No improvements were observed in the enjoyment scores	No adverse event occurred	Mean adherence 90% (44/49 min) wk-1, 83% (56/68 min) wk-2, 69% (45/65 min) and 43% (40/95 min) wks-3 and 4
*Studies on ADHD*						
Verret et al. [45]	3	Aerobic capacity, flexibility, muscular endurance, Gross motor skills	Behavioral problems, social competences; Attention functions response inhibition; Auditory sustained attention, divided attention	Increased muscular capacity (between groups). Motor skills, behaviour, and neuropsychological variables (information processing, and a better auditory, sustained attention)	Not reported	Not reported
Pan et al. [30]	6	Motor skills	Social behaviors and executive functions	Significant between groups differences in manual coordination, strength and agility, behavioral problems (social problems, attention problems, aggressive behaviors)	Not reported	89%
López-Williams et al. [46]	2	Strength/endurance, running speed	Social behavior and peer relationships	Both athletic performance and social behavior were significant predictors in the social acceptance of children with ADHD	Not reported	Not reported
O´Connor et al. [47]	4	Athletic competence and Motor Proficiency		Improvements (per-post and between groups) in sport knowledge and performance. Improvements (pre-post) in gross and fine motor skills	Not reported	Not reported
Pan et al. [49]	5	Aerobic capacity, flexibility, muscular endurance, Gross and fine motor skills		Significant between-group differences were observed in favor of EG in motor proficiency manual and body coordination, Strength and agility, bilateral coordination, and 20-m PACER. Non-significant changes were observed regarding abdominal and upper body muscle strength, balance and running speed and agility	Not reported	Not reported
Ziereis and Jansen [50]	4	Motor performance. Static and dynamic balance	Executive functioning. Working memory	Significant main effects (pre-post) in executive functions (digit-span, letter–number-sequencing, catching and aiming) and motor performance	Not reported	Not reported
Hupp and Reitman [51]	2	Dribbling and shooting test	Good sportsmanship, sport interest	No changes in dribbling or shooting ability but higher levels of interest in basketball and sportsmanship	Not reported	Not reported
Månsson et al. [52]	3		Inattention, hyperactivity, and impulsivity; emotional and behavioral functioning (prosocial behavior and positive attributes; HRQoL	Non-significant differences in symptoms (inattention, hyperactivity, and impulsivity). Significant improvement (pre-post) in parent-rated severity of ADHD symptoms and HRQoL. Significant between group improvement in reaction time variance, and fewer omission errors	No adverse event occurred	Not reported
Benzing and Schmidt [53]	6	Motor skills	Executive functions (inhibition, switching, updating); inattention, hyperactivity, and impulsivity	Exergame intervention group improved in specific executive functions (reaction times in inhibition and switching), general psychopathology as well as motor abilities compared to CG	Not reported	High dropout rate in the exergaming condition (n = 6)
Kadri et al. [54]	6		Cognitive function (attentional inhibitory control and sustained and selective visual attention)	Significant changes (pre-post) in all cognitive attention tests. Better cognitive performance in terms of selective attention than those in the control condition	Not reported	Not reported
*Studies on obesity*						
Walker et al. [55]	3		Self-esteem,Self-Perception, worries,	Body shape dissatisfaction significantly decreased and self-esteem improved. Global self-worth had increased by the end of the camp, as had athletic competence and physical appearance esteem	Not reported	Not reported
Cristian-Cosmin et al. [56]	4	Physical fitness (balance, running speed – agility, speed of limb movement, trunk strength, explosive power)		Significant improvement (pre-post) in fitness (balance, running speed – agility, speed of limb movement, trunk strength, explosive power) and body composition	Not reported	Not reported
Cvetković et al. [57]	5	Muscular fitness (lower-body power, change-of-direction speed, and flexibility), and cardiovascular fitness		Significant pre-to post improvements in lower-body power, flexibility, intermittent exercise and change-of-direction speed, and a significant lowering of maximal heart rate	Injury (n = 1)	Not reported
Cliff et al. [58]	4	Motor skills, lower-limb muscle strength	Perceived competence	Motor development, perceived athletic competence and perceived global self-worth significantly increased (pre-post)	Not reported	91%
Griffin et al. [60]	3		Enjoyment and Commitment	Significant difference in participants’ enjoyment of and commitment to physical activity	Not reported	Not reported
Jette et al. [61]	3	Physical work capacity test (V02 max was predicted)	Self-image Concept and personality	Decreases in resting and exercise heart rates and increases in physical work capacity. There were no measurable changes in personality assessment	Not reported	Not reported
Korsten-Reck et al. [62]	2	Physical performance (spiroergometry)		Improvements in performance capacity (Watt/kg BW)	Not reported	Not reported
Lofrano-Prado et al. [63]	5		Self-image Concept; symptoms of depression; binge eating; bulimia,; HRQoL	No between-group differences were observed for any of the assessed outcomes	Not reported	75%
Seabra et al. [64]	5	Cardiorespiratory fitness (VO2max)	Perceived psychological status; Self-esteem; self-perception; HRQoL	Participants improved (pre-post) in cardiorespiratory fitness, body image, self-esteem and quality of life; perceived themselves as more successful and physically competent; and were more attracted to participate (EG vs CG)	Not reported	> 85%
*Studies on cancer*						
Speyer et al. [65]	4		HRQoL	Physical functioning, role/social-physical, self-esteem, and mental health dimensions improved	Not reported	Not reported
Saultier et al. [66]	5	Aerobic capacity, flexibility, balance, upper and lower limb strength and muscle endurance	Self-esteem; HRQoL	Improved exercise capacity, self-esteem, and QoL. Improvements (pre-post) in exercise capacity, flexibility, balance, upper limb strength, and abdominal muscle endurance. Self-esteem change was similar in both groups. Significant between-group differences in HRQoL	No adverse event occurred	Not reported
Hamari et al. [67]	5	Motor skills		No differences between the intervention and control group in motor performance	No adverse event occurred	Not reported
Howell et al. [68]	4	Handgrip strength, lower-limb strength	Neurocognitive General intelligence, Executive Function, HRQoL	Significant (pre-post) improvements in hand grip strength, number of sit-ups and pushups, neurocognitive function, and HRQoL outcomes improved in the intervention, but not in CG	Not reported	Not reported
Li et al. [69]	8		Self-efficacy, HRQoL	EG showed statistically significantly higher levels of self-efficacy, and better HRQoL than the control group at 12 months	Not reported	Not reported
*Study on asthma*						
Westergren et al. [71]	2	Lung function, Cardiorespiratory fitness	HRQoL	Significant pre-post differences in HRQoL, no changes in cardiorespiratory fitness were reported	Not reported	90%
*Study on cystic fibrosis*						
Hakim et al. [72]	4		HRQoL	HRQoL (physical, emotional, social dimensions) did not show significant differences	Not reported	Not reported

The PEDro scale (item 1, pertaining to external validity, is excluded from the total score calculation, which ranges from 0 to 10). *HRQoL* Health-related quality of life

The psychosocial outcomes were categorized into three distinct groups. The first group, labeled "Behavioral Problems and Social Competencies", focused on examining the interplay between behavioural problems and social competencies. These variables aimed to investigate the relationship between different aspects of behaviour and social functioning, encompassing social skills, social behaviour, and psychological factors. The variables within this group encompassed measures of social behaviours, social skills, and peer relationships, as well as psychological factors such as anxiety, depression, and self-perception. Additionally, specific variables related to ASD and ADHD were included to explore the severity of their behaviours and their impact on social functioning. Social behaviours and skills (behavioural problems and social competencies, or communication skills) were assessed in 13 (25%) studies [25, 26, 30–32, 45, 46, 48, 59, 73–76]. Other psychological included factors such as self-image concept and personality [35, 53, 55, 58, 61, 63, 64, 66, 75] or perceived competence [58, 70]. Another group of variables focused on Executive Functioning and Attention Abilities. Multiple domains of attention, including selective attention, sustained attention, attentional control, and divided attention. The last group focused on Health-Related Quality of Life (HRQoL), that was assessed in 13 occasions [32, 39, 40, 43, 44, 52, 63, 64, 66, 68, 69, 71, 72]. An overview of the tools utilized for the assessment of these outcomes is available in Supplementary File 2.

### ASD

The overall methodological quality of the included studies, as assessed by the PEDro scale ranged from 1 to 8 out of 10. Notable studies such as Pan et al. [74] achieved a high score of 8, reflecting excellent methodological quality, while the studies by García-Gómez et al. [25] and López-Diaz et al. [26] received lower scores, suggesting lower methodological quality.

The interventions for individuals with ASD encompassed activities such as horse-riding, soccer, judo, ball games, dances, active video games, swimming, and table tennis [25–29, 31, 32, 37, 48, 59, 70, 73–76]. Two of the studies also employed active video games [28, 70]. The interventions varied in terms of activity type, duration, and frequency, reflecting the diversity of approaches to engage this population in physical activities and sport. The duration of interventions ranged from a few weeks to several months. One of the studies spanned one week [75], while most interventions spanned 8–14 weeks with sessions lasting 45–75 min conducted once or twice a week. A more intensive approach involved a 12-week program with 40-min sessions conducted five times a week [32, 73].

These studies on sports interventions for CaA with ASD explored a range of physical and psychosocial outcomes, providing valuable insights into the potential benefits of such interventions. The evaluation of fitness changes was conducted in three interventions [28, 29, 32] encompassing assessments of muscle endurance, running speed and agility, balance, bilateral coordination, flexibility, and strength. Non-significant changes in these outcomes were reported across these interventions. Motor skills were evaluated in nine studies [26, 28, 29, 31, 32, 48, 59, 70, 75], with ball games and sports leading to significantly enhanced motor skills, particularly object-control skills, among the participants [48, 59]. Conversely, an intervention, which utilized Kinect Sports as its foundation, targeted gross motor skills and perceived competence. Although there were no significant enhancements in actual skills, participants reported improved perceptions of competence [70]. Aquatic skill measures and social behavior were evaluated, showing significant social improvements alongside improvements in aquatic skills [74]. Similarly, enhancements in swimming skills were examined in another study [27].

Improvements in executive functioning and attention abilities were reported in four studies [28, 31, 73, 76]. Significant enhancements in working memory, inhibition, regulation, social communication, and repetitive behavior were observed [73], alongside sustained improvements in motor skill proficiency and executive function for at least 12 weeks [31]. Similarly, various aspects including adaptive skills, social skills, leadership, withdrawal, anxiety, depression, behavioral problems, atypicality, aggressiveness, hyperactivity, attention problems, and somatization were assessed, noting significant improvements in aggressiveness, interpersonal relations, and social inclusion [25]. Autism-related behaviors and social communication difficulties were also examined, identifying significant enhancements in repetitive behaviors, social interaction, social communication, and emotional response [37].

### Cerebral Palsy

The PEDro scale revealed excellent methodological quality in the study conducted by Chiu et al. [34] with a high score of 9. Other examples with a good methodological quality (i.e., score of 8) were found [35].

CaA with cerebral palsy participated in interventions including golf training, home-based active video games, and participation in soccer, netball, T-ball, cricket, swimming, tennis, dance, martial arts, basketball, soccer, baseball, and adaptive cycling [33–35, 38–44]. Home-based Wii Sports Resort training, and Virtual Reality Games were also employed in four studies [33, 34, 39, 41].

Intervention periods ranged from 4 weeks to 1 year, with different session frequencies and durations. Among the included studies, the intervention period varied from 4 [39, 44] to 12 [33, 34] weeks, with sessions lengths of only 25 min [39] to 30 min [41] and frequencies varying from 1 to 5 times per week. Notably, there were longer-term studies such as Feitosa et al. [43], with a duration of 1 year and 156 sessions.

Among the studies assessing physical fitness in CaA with cerebral palsy [33–36, 38, 40, 43], several reported significant improvements in various domains. Significant differences were observed in gross motor skills, walk, and balance [42]. Enhancements in physical competence, walking, running, jumping, throwing, and sports participation were also documented [40]. Similar improvements were reported in another study, which documented gains in mobility, cardiorespiratory fitness, flexibility, balance, walking speed, and gross motor skills [38]. Clinically significant improvements were highlighted in functional mobility, physical activity competence, and strength [36]. Similarly, positive impacts were observed on gross motor skills, aerobic capacity, and lower limb strength [35]. However, not all studies observed significant changes. For example, six weeks of home-based Wii™ training plus usual therapy did not improve coordination, strength, or hand function in children 9.5 ± 1.9 years of age [34]. Further, eight weeks of a video-game based therapy did not enhance manual dexterity for carrying out everyday tasks in children aged 7.0 ± 1.9 years [41].

In the domain of motor skills assessment, while non-significant differences were noted in gross motor skills [42], improvements were highlighted in motor functions of upper limb extremities, including increased quantity of limb movements, following virtual reality intervention [41]. Reaction time measures significantly improved in the experimental group, suggesting positive effects on motor skills [39]. Additionally, significant gains in mobility and gross motor skills were documented [38], along with positive impacts observed on gross motor skills [35]. These authors also reported positive effects in several domains, including self-efficacy, goal achievement, child perceptions of goal performance, and satisfaction. The study found that sports skills training had an effective impact on promoting advanced motor skill gains in CaA with cerebral palsy.

Finally, quality of life (HRQoL) was assessed in four studies [39, 40, 43, 44]. Significant improvements were demonstrated in self-identified sports-focused participation and activity level goals, reflecting a positive influence on the perceived HRQoL of the participants [36]. Additionally, significant improvements in dimensions related to mobility and physical function were observed [43, 44], collectively contributing to a positive biopsychosocial profile and, consequently, improved HRQoL.

### ADHD

In terms of methodological quality, Benzing and Schmidt [53] and Kadri et al. [54] each scored 6, indicating good methodological quality. At the lower end, studies such as López-Williams et al. [46] and Hupp and Reitman [51] scored 2, suggesting a lower methodological quality. These findings provide an overview of the diverse methodological rigor observed across studies of attention-deficit/hyperactivity disorder within our review.

For individuals with ADHD, interventions involved various sports and physical activities such as basketball, soccer, taekwondo, tag and ball games, table tennis, horse-riding, and target-shooting sports [30, 45–47, 49–54]. These studies employed diverse sports/physical activity modalities to assess the effects on ADHD symptoms and overall well-being. Notably, the durations of interventions ranged from 3 weeks [51] to 1.5 years [54], with frequencies spanning from 1 to 5 times per week.

The studies examining physical fitness outcomes in individuals with ADHD demonstrate diverse effects of physical activity interventions. Specifically, increased muscular capacity and enhancements in aerobic capacity, flexibility and muscular endurance were reported [45], together with improvements in strength/endurance and running speed [46]. These changes were associated with improvements in athletic competence and motor proficiency [47]. Additional positive effects on motor performance, static and dynamic balance were observed [50]. The studies investigating motor skills outcomes reported improvements in gross [30, 45, 49, 53] and fine motor skills [49] following a comprehensive exercise intervention.

Various studies investigated cognitive and executive functions [30, 50, 52–54].

Improved cognitive performance, particularly in selective attention [54] or working memory [50], was observed following the intervention, with enhancements in specific executive functions, such as reaction times in inhibition and switching, as well as general psychopathology and motor abilities [53]. Conversely, Månsson et al. [52] found non-significant beneficial effects on inattention, hyperactivity, and impulsivity but reported significant improvements in emotional and behavioral functioning. The studies collectively suggest that exercise interventions may positively impact cognitive and executive functions in individuals with ADHD.

### Obesity

The methodological assessment for studies related to obesity in our review reveals overall a moderate methodological quality, while the studies by Walker et al. [55], Griffin et al. [60], and Jette et al. [61] each scored 3, and Korsten-Reck et al. [62] scored 2, suggesting lower methodological quality. Overall, the results indicate diverse methodological rigor across the obesity studies included in our review.

The interventions for individuals with obesity included volleyball, football, and physical education activities such as swimming and balloon volleying [55–58, 60–64]. Only nine articles have examined the effects of a sports program on this population. Of these, five have utilized team sports [56, 57, 61, 63, 64], two have employed group-based sports activities [55, 58], and three have opted for mixed interventions alternating between group sports/team sports or individual sports activities [60, 62, 63]. The programs implemented varied in duration, frequency, and activity type. The interventions’ duration ranged from 4 to 24 weeks, although most of them were ≥ 10 weeks and < 24 weeks.

Significant improvements were noted in physical fitness domains such as balance, running speed-agility, change-of-direction speed, as well as cardiorespiratory fitness. Motor skills and lower-limb muscle strength also demonstrated significant enhancements. However, not all outcomes were improved; Cvetković et al. [57] found significant pre-to-post improvements in change-of-direction speed, as well as cardiovascular fitness, but not in in muscular fitness, lower-body power or flexibility. Muscle strength was not improved in the study conducted by Cliff et al. [58].

In the current review, psychosocial outcomes were assessed in 6 studies, overall showing improvements in several indicators of health status (e.g., self-esteem improved) and perceived competence [55, 58, 64]. These interventions yielded positive changes in self-esteem, body image, and overall quality of life. However, it is noteworthy that certain outcomes, such as personality and perceived psychological status, did not exhibit significant changes following the interventions.

### Cancer

Only five studies have focused on CaA with cancer [65–69]. The methodological evaluation reflected an excellent methodological quality in the study by Li et al. [69], which achieved a high score of 8. A moderate methodological quality was also achieved in other studies [66, 67]. Nevertheless, despite the low number of studies, all of them were randomized trials and, the average number of participants in the experimental groups across these studies was 57.

CaA with cancer engaged in various activities like ball games, racket sports, fighting activities, dance, basketball, badminton, yoga, skiing, swimming, paddling, climbing and active video games [65–69].

Regarding the settings, one study was conducted in the hospital [65], two combining hospital and home [67] or outdoor activities [66]. Another study was conducted only in the home [68] and the remaining study organized a 4-day physical activity program for cancer patients, involving four sessions during their stay at the campsite [69]. A diverse range of physical activity programs for cancer patients lasting less than 4 weeks were implemented [65, 66] Active video games were implemented for 8 weeks, both during hospitalization and at home, in another RCT [67]. Additionally, an interactive website was used to promote physical activity in a 24-week intervention, with the frequency being voluntary and the duration not specified [68].

Only two studies assessed fitness in CaA with cancer [66, 68] reporting improvements in aerobic capacity, flexibility, balance, upper and lower limb strength and muscle endurance. No differences in motor performance (motor skills) were observed in the Hamari et al.’s study [67] focused on the impact of active video games, specifically Nintendo WiiFit™ games, on cancer patients. The active video game sessions were conducted five times a week for a duration of 30 min each, totaling 56 sessions over an 8-week period.

Positive results in physical, social, and psychological aspects were achieved combining different sports [65]. Exercise capacity, self-esteem, and HRQoL were also improved in another study [65]. Two additional studies assessed HRQoL as well [68, 69].

### Asthma

Only one study examined the effect of sports participation interventions in CaA with asthma, and that study had a low methodological quality (2 out of 10 on the PEDro scale) [71]. The study conducted by Westergren et al. [71] was a relatively small-scale investigation involving 6 participants. The intervention consisted of active play sessions, including activities such as ball and team games. The duration of the intervention spanned 6 weeks, with sessions held twice a week, each lasting 60 min. The study aimed to assess the impact of regular active play on individuals with asthma, potentially improving the perception of fitness and overall well-being.

### Cystic Fibrosis

For cystic fibrosis, only one study was included [72]. Different activities were performed (cycling, swimming, walking, dancing, playing ball, skipping ropes, jumping, upper extremity stretching, exercises involving the trunk and lower extremities in gymnastics) over one week resulting in improvement in physical function. The duration of the intervention spanned one week, with participants engaging in sessions lasting 30 to 45 min, conducted four times a week.

### Adverse Events and Adherence to the Interventions

Adverse effects data were available in only nine studies (one in ASD, four in cerebral palsy, one in ADHD, one in obesity, and two in cancer). The majority of the interventions examined showed no observable adverse effects; however, in the field of obesity, a single study [57] reported an injury in one participant, with no additional data provided. Notably, a study by Clutterbuck et al. [40] of CaA with cerebral palsy noted some activity modifications that were tailored to individual participants’ capabilities. However, beyond this specific instance, no detrimental outcomes were reported across the interventions reviewed.

Alongside the absence of noticeable adverse effects, information regarding adherence was available in only fourteen studies. These studies consistently reported favorable adherence rates to the investigated interventions. Adherence to interventions in ASD exhibits a varied range of 79–100%, underlining the presence of variability but generally high adherence within the examined sample. Notably, only 4 out of 15 studies provided data on adherence. Contrastingly, in cerebral palsy, adherence data were available from 5 out of 11 studies, with reported levels exceeding 75% [36] or surpassing 90% [35, 77]. The study conducted by Lai et al. [44] showed fluctuating adherence levels throughout the intervention. In the initial week, there was a notable mean adherence of 90%. However, adherence declined in the second week to 83% (56 out of 68 min), and further reductions were observed in weeks three and four, with adherence rates dropping to 69% (45 out of 65 min) and 43% (40 out of 95 min), respectively. For ADHD, data on adherence were only reported in one study [30]. In the context of obesity, adherence to interventions exceeded 75%, as reported by three studies. The lone study on asthma reported a commendable adherence rate of 90% [71]. Notably, no adherence data were reported for interventions in cancer or cystic fibrosis.

## Discussion

This systematic review aimed to examine the effects of sports interventions on physical and psychosocial outcomes in various populations, including individuals with ADHD, cerebral palsy, ASD, cancer, asthma, cystic fibrosis, and obesity. The review found that sports interventions encompassing a wide range of activities, such as basketball, soccer, swimming, and active video games, yielded positive outcomes in terms of motor performance, physical fitness, social behaviours and skills, psychological factors, executive functioning and attention abilities, and HRQoL. However, the diverse results observed underscore the necessity for personalized approaches in therapeutic interventions. While there are many different approaches yielding varied results, standardization within and across different CaA groups is crucial for better understanding the effects. The review acknowledges the importance of tailoring interventions based on specific conditions, age, abilities, and desired outcomes, emphasizing the need for personalized approaches to optimize effectiveness and address individual needs effectively.

Still, overall, sports interventions showed promising benefits in improving the target populations’ physical and psychosocial outcomes. Despite the promising results, youth with chronic diseases often fail to meet the guidelines for healthy physical activity. Only 26% of youth with chronic diseases participate in sports once a week [78]. This means they engage in competitive and recreational sports even less frequently than their healthy peers. Various barriers can explain these differences, including both personal (e.g., attitudes from the parents or teachers) and environmental factors (e.g., transportation to an adapted sports facility) [79].

### ASD

In patients with ASD active video games offer a multisensory and interactive experience which could improve motivation to participate in physical activities. Compared with sports and active recreation activities [28], exergaming (Kinect) improved motor function in CaA with ASD. However, the authors reported that these interventions might not sufficiently promote correct movement patterns to influence skills in CaA [70].

Important improvements in executive functioning and attention abilities were reported [28, 30, 31, 73, 76]. Possible explanations for these improvements include the involvement in structured rules and routines, which can help develop cognitive skills (i.e., components of executive functioning) [80]. Further, the requirement of concentration and focus can also enhance attention skills [25]. Notably, it is essential to emphasize that only two of the five studies reported above [31, 76] integrated interventions involving team sports. Nevertheless, it is noteworthy that engagement in team sports can also foster social interaction and cooperation, potentially exerting a positive influence on executive functioning and attention capabilities [73].

### Cerebral Palsy

CaA with cerebral palsy, with moderate to severe learning disabilities encounter limited access to physical activity and sports [81]; consequently, the most frequently performed physical or sports activities were based on smart devices, specifically video games [33, 39, 41, 77], as well as video dancing [44]. This highlights the growing utilization of technology in promoting physical activity in this population to increase motivation [82]. Motivation was identified as a significant factor influencing adolescents’ participation in physical activity [83]. However, these interventions’ physical and psychosocial benefits must be considered with caution as some studies did not report positive results [41, 77].

There could be several reasons why video game-based therapy in CaA with cerebral palsy may not improve physical or psychosocial outcomes. Some potential reasons include a lack of specificity, as these interventions may not specifically target the underlying motor impairments and functional limitations associated with cerebral palsy. The games used might not adequately address the specific movement patterns or motor skills that need to be improved [84]. Another important reason can be insufficient dosage. The frequency and duration, and intensity of video game-based sessions may not be sufficient to induce meaningful changes in motor abilities. Individual differences can also partly explain the lack of significant differences in some outcomes.

We provided evidence that sports interventions can significantly contribute to better motor function and physical fitness, which is particularly important in youth with a neuromuscular disorder [85]. Due to decreased coordination, these children typically required additional time to perform motor tasks compared to their peers without such impairments. Children with cerebral palsy can have diverse presentations and varying levels of motor impairments (i.e., spasticity, dyskinetic or/and ataxic), with abnormalities of coordination and balance [86]. Video game-based interventions may only cater to some of the individual needs and abilities, leading to limited effectiveness across the population. Lastly, limited transfer to real-life activities might also be an influencing factor as the movements and coordination required in video games may not directly translate into improved abilities for real-world tasks.

The findings of one study [81] identified four categories of factors that influence participation in physical activity: (1) Musculoskeletal pain and other; (2) Knowledge, exercise skills, and life skills such as problem-solving, decision-making, planning, and organizing; (3) Availability challenges, including lack of transportation, professional guidance, adapted and community-based programs, and enjoyable activities; (4) Social support from professionals and peer support with opportunities for social interaction. Therefore, in contrast with the more severely affected children, those with mild to no learning disabilities utilized physical activity to manage pain and maintain functional abilities. In the current study, multiple types of sports activities were used as individual sports (e.g., golf or swimming) and team sports (e.g., Soccer, netball). These interventions promoted motor skill gains and sports-specific physical competence, improved fitness, mobility, and global function, and favoured enjoyment and HRQoL. Sports can, therefore, play a crucial role in overcoming barriers to physical activity in CaA with cerebral palsy by addressing musculoskeletal pain and other impairments [81]. Sports have contributed to the development of knowledge and exercise skills, and by offering enjoyable activities, these activities can enhance the attractiveness and accessibility of physical activity for these CaA. However, some considerations must be highlighted. In this population group professional guidance is needed to cater the adapted sport-based programs to the specific needs of children with cerebral palsy [40]; coaches should tailor the sport interventions to accommodate individual capabilities and ensure a safe and inclusive environment. Further, coaches should align the sport interventions with the child’s functional goals. These goals may include improving motor skills, coordination, balance, flexibility, and physical fitness. Finally, coaches should foster positive social interactions among participants and encourage peer support. Providing CaA with cerebral palsy opportunities to collaborate and participate alongside their typically developing peers can enhance their overall experience [87]. In the current review, coaches prioritized the safety of the participants. They were aware of the specific risks associated with cerebral palsy and took appropriate precautions to minimize the risk of injury during training and competitions.

### ADHD

Individuals diagnosed with ADHD often experience social isolation and rejection due to limited motor skills development, lack of coordination, and diminished attention and executive functioning abilities [45]. Sports activities offer unique physical, psychological, and social advantages, distinguishing them from traditional exercise interventions [23, 88]. Given the characteristics of individuals with ADHD, sports can be viewed as a promising tool with significant potential to address their needs. This perspective is supported by the findings of the nine identified articles on sports programs targeting individuals with ADHD in the current review. These studies reveal improvements in physical fitness, motor skills, and variables related to sports performance [30, 45–47, 49–53], as well as enhancements in psychosocial variables [30, 45, 46, 50–53]. However, despite these positive findings, further studies with robust designs and methodologies are needed, with longer durations, as most of the existing research does not exceed three months. Additionally, it is worth noting that there were some inconsistent results in the existing literature, highlighting the importance of more comprehensive investigations into the effects of physical activity/sports interventions in individuals with ADHD.

The articles found exhibit a high degree of heterogeneity regarding the type of sport. This limitation prevents the recommendation of a specific sport for this population. However, many of the identified sports share the common element of teamwork or group participation [30, 45–47, 50, 51]. This is important because children with ADHD often have problems with peer relationships and are at increased risk for long-term deficits in social functioning [46]. Additionally, some of these studies simultaneously included multiple sports activities in the intervention [45–47, 50, 53]. This may be beneficial, as evidence suggests that interventions incorporating multiple activities may have the most significant potential to improve ADHD symptoms [53]. The weekly frequency of the sessions also varied substantially across the different studies. Although there appear to be some benefits with only one session per week [49, 50, 52], this needs to be examined in more detail. Similarly, the duration is also an aspect that needs to be clarified. Nevertheless, it is worth noting that the only RCT among the nine articles reported benefits with just three sessions per week, lasting 30 min each, over a period of 2 months [53]. These authors propose exergaming as an alternative to traditional physical activity programs, as children with ADHD often find them less interesting and exhausting, limiting their engagement [89].

### Obesity

Despite the many existing studies on physical activity in CaA with obesity [90], the diverse range of sports within this group complicates the formulation of recommendations regarding the most suitable type of sports intervention for treating obesity in this age group. CaA with obesity sometimes experience rejection from their peers due to their lack of physical activity skills [91]. Consequently, it is important to offer alternatives based on sports programs or non-competitive games as motivating practices for this population, as these ages are crucial for developing obesity and adherence to physical activity.

Overall, it is noteworthy that the duration of the interventions tends to be relatively short (< 24 weeks). According to a systematic review that studied the duration of interventions for reducing overweight and obesity in CaA, the duration of the studies may not be adequate, as they recommend at least ten months (40 weeks) to avoid rebound effects and, therefore, achieve positive medium-to-long-term results [92]. Undoubtedly, the duration of interventions can influence the outcomes, as well as the adherence to them. Therefore, finding a balance will be necessary.

### Cancer

Only five studies have focused on CaA with cancer [65–69]. The settings where the studies were conducted reflect the unique situation in this population, as CaA with cancer combine long periods in the hospital with treatments at home. Thus, we found one in-hospital study [65], one home-based study [68], and three mixed settings [66, 67, 69]. Due to the characteristics of this condition, where the immune system can be affected, the possibility of offering sports programs through exergames can be an alternative. The findings in this regard are controversial, as one study did not find differences between the intervention and control groups in physical outcomes [67]. Similarly, in the study by Howell et al. [68], the intervention was delivered through an interactive website designed to encourage physical activity through rewards. Such alternatives and interventions are supported by the growing body of knowledge that uses gamification and new technologies to promote healthy lifestyles with promising results [93]. Given the long hospitalization periods in this population group, establishing in-hospital programs is crucial.

### Asthma

Only one article was identified in patients with asthma [71]. Significant improvements in lung function, cardiorespiratory fitness and quality of life were reported in this twice weekly (6 weeks) intervention based on the ball and team games. ParticipaPts in the study were involved in different focus groups, and they could express that their daily lives and participation in physical activity were constrained by asthma (e.g., they became rapidly exhausted). Reasons limiting their adherence were also described. On the one hand, the authors highlighted the importance of instructors in creating enjoyable programs, enhancing the children’s normality and independence, and on the other, the importance of mutual support between participants, which may let them feel normal and competent.

### Cystic Fibrosis

Only one study, involving different physical activities, was identified in cystic fibrosis [72]. Despite the improvement in physical function, the short duration of the program (one week) and the control of variables mean caution is required when interpreting the results. The authors believe that these sports activities can potentially improve these patients’ quality of life.

### Adverse Events and Adherence to the Interventions

While previous literature has reported the absence of adverse effects in sport-related activities for CaA with chronic diseases [94, 95], the limited availability of data in our study prevents us from drawing definitive conclusions. The paucity of information emphasizes the need for further research and comprehensive data collection to provide a more comprehensive understanding of potential adverse effects in the context of these interventions. It is important to acknowledge the study of Clutterbuck et al. [40] on children with cerebral palsy, which observed activity modifications accommodating individual capabilities, demonstrating a proactive approach to ensure participant safety. However, lack of available data limits our ability to definitively confirm their safety profile.

Regarding adherence rates, the few studies with available data in the current review are in line with previous evidence reporting positive adherence outcomes in sport-related interventions for CaA with chronic diseases, reporting average adherence rates, regardless of condition (e.g., cancer, cardiovascular disease, and diabetes), of 77% [96]. However, it is important to consider the study conducted by Benzing and Schmidt on ADHD [53], which specifically investigated the exergaming condition and reported a relatively high dropout rate. This finding echoes previous literature highlighting the potential challenges in maintaining adherence to certain interventions, particularly in specific populations such as individuals with ADHD. As reported above, active video games (i.e., exergaming) have been shown to be beneficial for CaA in clinical and rehabilitative settings [97]. Data from the literature indicate that these interventions may offer an interesting impact on childhood with chronic diseases; nonetheless, there are also potential challenges in maintaining adherence to these interventions. When compared to sport-related activities, active video games often involve solitary gameplay, which may limit social interaction. This is particularly important in the current review, where social support was crucial in maintaining adherence to exercise programs [30, 58, 76]. Without opportunities for social engagement or competition, individuals may feel less motivated to continue with exergaming [53]. Similarly, the study by Lai et al. [44] on individuals with cerebral palsy, utilizing video and music as intervention components, reported varying adherence rates throughout the intervention period. While the adherence rates were initially high, they gradually declined over time. This finding is consistent with previous research that has acknowledged the potential fluctuations in adherence levels in long-term interventions. It emphasizes the importance of continuous monitoring and adaptation of intervention strategies to sustain engagement and adherence over extended periods [98]. This was also the case of the obesity interventions in the current review [58, 63, 64], despite the high level of dedication and commitment among individuals undergoing the interventions, it was supported the notion that tailored approaches can effectively promote adherence and positive outcomes in weight management programs [99].

Overall, this review aligns with and builds upon previous literature, emphasizing the limited reporting of adverse effects and the favorable adherence rates observed in a small subset of studies on CaA engagement in sport-related activities among individuals with chronic diseases. They highlight the need for continued research and the development of tailored interventions to ensure participant safety and optimize adherence in this population. In the present systematic review, various factors appear to have influenced the observed outcomes. Methodological quality of the studies, the characteristics of the study population, program duration, frequency, and particularly, the nature of the activities conducted, seem to have played significant roles. While these factors have been individually described for each pathology, some general conclusions can be drawn across all groups. Shorter interventions (< 8 weeks) tend to yield more modest results compared to longer-duration interventions [38, 39, 51, 59, 67, 71, 72]. Additionally, interventions based on active video games [33, 39, 67, 70] or other activities with less social interaction [52] tend to exhibit comparatively fewer significant changes than interventions based on traditional sports activities or more recreational pursuits.

### Study Limitations

The systematic review has several limitations. Firstly, a wide range of chronic diseases are included in the review, which may introduce heterogeneity in terms of the underlying pathophysiology, treatment approaches, and individual needs. Additionally, the age range of the participants is broad, spanning from early childhood to adolescence, which further adds to the variability in terms of developmental stages and potential differences in intervention outcomes. Moreover, the review encompasses a diverse array of sports and physical activities, making it challenging to draw definitive conclusions regarding the specific benefits of each discipline. Another limitation pertains to using various assessment instruments across studies, which introduces variability in outcome measures and may impact the comparability of results. In addition, it is important to acknowledge that the available literature for this review provided limited information on adverse effects and adherence rates. The scarcity of data in these domains across the selected studies is a notable limitation, and further research with a specific focus on these aspects is warranted to enhance our understanding of comprehensive outcomes in this context. Despite these limitations, the systematic review provides valuable insights into the potential effects of sports interventions in CaA with chronic diseases, serving as a foundation for future research and intervention development.

## Conclusion

Sports interventions promote motor skill gains, sports-specific physical competence, fitness, mobility, global function, enjoyment, and quality of life among CaA with chronic diseases. The reviewed interventions demonstrated a relative safety profile with no reported adverse effects. Adherence rates were generally favourable, with high levels of engagement and commitment observed. Challenges in maintaining adherence were noted in certain interventions, particularly in populations such as individuals with ADHD. Tailoring interventions to individual capabilities, providing professional guidance, and fostering positive social interactions were highlighted as important considerations in maximizing the benefits of sports interventions on physical and psychosocial outcomes in CaA with chronic diseases. Nevertheless, further research is needed to better understand the specific determinants of these interventions, including frequency, duration, or type, and to establish the positive associations between sports participation and the overall well-being of CaA with chronic diseases. These results provide valuable insights for practitioners, coaches, and athletes alike, promoting confidence in implementing these interventions as part of comprehensive training and performance enhancement programs without significant concerns regarding adverse effects.

### Supplementary Information

Additional file 1. 

Additional file 2.

## Data Availability

The datasets used and/or analysed during the current study are available from the corresponding author on reasonable request.

## Abbreviations

ADHD: Attention-deficit/hyperactivity disorder
ADHD-RSIV: Attention-deficit/hyperactivity disorder
ASD: Autism spectrum disorders
BASC: Behavior assessment system for children
BOT-2: Test of motor proficiency second edition
BPFT: Brockport physical fitness test
BSQ: Body shape questionnaire
CaA: Children and adolescents
CBCL: Child behavior checklist
CBTT: Corsi block tapping task
CDC: Centers for disease control and prevention
CHEXI: Childhood executive functioning inventory
CHQ: Child health questionnaire
CP QOL-Child: Cerebral palsy quality of life questionnaire
CSAPPA: Children’s self-perceptions of adequacy in and predilection for physical activity
CVD: Cardiovascular diseases
CY-PSPP: Children and youth physical self-perception profile
D–KEFS: Delis–Kaplan executive function system
EPI: Eysenck personality inventory
GARS-3: Autism rating scale-third edition
GAS: Goal attainment scaling
GHQ: General health questionnaire
GNG: Go/No-Go
HRQoL: Health-related quality of life
MMPI: Minnesota multiphasic personality inventory
MPST: Muscle power sprint test
MVPA: Moderate to vigorous physical activity
PACER: Progressive aerobic cardiovascular endurance run
PAQLQ: Asthma quality of life questionnaire
PedsQL: Pediatric quality of life inventory
PMSC: Perceived movement skill competence
PODCI: Pediatric outcome data collection instrument
PSI: Physical self-inventory
RBS-R: Repetitive behavior scale-revised
RCT: Randomized controlled trials
RT: Randomized trials
SCQ: Social communication questionnaire
SDQ: Strengths-and-difficulties-questionnaire
SPPC: Self-perception profile for children
SRS-2: Social responsiveness scale
SSBS-2: School social behavior scales
SSIS: Social skills improvement system
SWRIS: Weight-related issues scale
Tea-Ch: Test of everyday attention for children
TGMD: Test of gross motor development
VABS-2: Vineland adaptive behaviour scales
WCST: Wisconsin card sorting test
