# Supplementary material for: Physical and Psychosocial Benefits of Sports Participation Among Children and Adolescents with Chronic Diseases: A Systematic Review
Source: Sports Med Open. 2024 May 15;10:54. doi: 10.1186/s40798-024-00722-8 (PMC11096140; doi:10.1186/s40798-024-00722-8)
Supplement: Supplementary file 1 — Additional file 1. [file 40798_2024_722_MOESM1_ESM.docx]

**Supplementary File 1. Search strategy**

PUBMED

( child* OR kids OR boys OR girls OR young* OR adolescents OR youth ) AND ( ( sport* OR physical AND activ* OR leisure-activities OR athlet* OR team OR exercise OR player* ) AND ( obes* OR asthma OR diabet* OR haemophilia OR cvd OR cancer OR cystic AND fibrosis OR epilepsy OR developmental AND disabilities OR cerebral AND palsy OR autism AND spectrum AND disorders OR attention-deficit OR hyperactivity AND disorder OR adhd OR post-traumatic AND diseases ) )

Limiters: <18 years; Human

SCOPUS

( TITLE ( children  OR  kids  OR  boys  OR  girls  OR  young  OR  adolescents  OR  youth ) )  AND  ( TITLE ( sport  OR  physical-activity  OR  leisure-activities  OR  athlete  OR  team  OR  exercise  OR  player ) )  AND  ( TITLE ( obesity  OR  asthma  OR  diabetes  OR  haemophilia  OR  cvd  OR  cancer  OR  cystic  AND  fibrosis  OR  epilepsy  OR  developmental-disabilities  OR  cerebral-palsy  OR  autism-spectrum-disorders  OR  attention-deficit  OR  hyperactivity-disorder  OR  adhd  OR  post-traumatic-diseases ) )

SportDiscus

TI ( ( obese or obesity ) OR asthma OR diabetes OR haemophilia OR ( cvd or cardiovascular disease ) OR cancer OR cystic fibrosis OR epilepsy OR ( developmental disabilities or autism ) OR cerebral palsy OR ( attention deficit hyperactivity disorder or adhd ) OR ( post traumatic disorder or ptsd ) ) AND TI ( sports OR physical activity OR leisure activities OR athletes OR team OR players OR exercise ) AND TI ( ( children or adolescents or youth or child or teenager ) OR kids OR ( boys or girls ) )

CINAHL

#s1 ( children or adolescents or youth or child or teenager ) OR kids OR ( boys or girls )

#s2 sports OR physical activity OR leisure activities OR athletes OR team OR players OR exercise

#s3 ( obese or obesity ) OR asthma OR diabetes OR haemophilia OR ( cvd or cardiovascular disease ) OR cancer OR cystic fibrosis OR epilepsy OR ( developmental disabilities or autism ) OR cerebral palsy OR ( attention deficit hyperactivity disorder or adhd ) OR ( post traumatic disorder or ptsd )

Web of Science

((TI=(child* OR kids OR boys OR girls OR young* OR adolescents OR youth)) AND TI=(sport* OR physical AND activ* OR leisure-activities OR athlet* OR team OR exercise OR player*)) AND TI=(obes* OR asthma OR diabet* OR haemophilia OR cvd OR cancer OR cystic AND fibrosis OR epilepsy OR developmental AND disabilities OR cerebral AND palsy OR autism AND spectrum AND disorders OR attention-deficit OR hyperactivity AND disorder OR adhd OR post-traumatic AND diseases )

Psycinfo

[(child$ OR boys OR girls AND kids AND young AND Adolescents AND Youth) AND (Sport OR (Phisical activity) OR (leisure activity) AND athlete AND Team AND Exercise AND Player) AND (obesity OR asthma OR diabetes OR haemophilia OR cvd OR cancer OR cystic AND fibrosis OR epilepsy OR developmental AND disabilities OR cerebral AND palsy OR autism AND spectrum AND disorders OR attention-deficit OR hyperactivity AND disorder OR adhd OR post-traumatic AND diseases)](https://www.proquest.com/recentsearches.recentsearchtabview.recentsearchesgridview.scrolledrecentsearchlist.checkdbssearchlink:rerunsearch/CEC6F4CAE71146ABPQ/None?site=psycinfo&t:ac=RecentSearches)

ERIC

1 (children or kids or boys or girls or young or adolescents or youth).af. 406847

2 (((sport or physical) and activity) or leisure-activities or athlete or team or exercise or player).af. 66127

3 ((((((((((((obes* or asthma or diabet* or haemophilia or cvd or cancer or cystic) and fibrosis) or epilepsy or developmental) and disabilities) or cerebral) and palsy) or autism) and spectrum and disorders) or attention-deficit or hyperactivity) and disorder) or adhd or post-traumatic) and diseases).af. 135

4 1 and 2 and 3 4

5 (obesity or asthma or diabetes or haemophilia or (cvd or cardiovascular disease) or cancer or cystic fibrosis or epilepsy or developmental disabilities or cerebral palsy or (attention deficit hyperactivity disorder or adhd) or (post traumatic disorder or ptsd)).af. 26755

6 1 and 2 and 5 1704
