# Supplementary material for: Physical and Psychosocial Benefits of Sports Participation Among Children and Adolescents with Chronic Diseases: A Systematic Review
Source: Sports Med Open. 2024 May 15;10:54. doi: 10.1186/s40798-024-00722-8 (PMC11096140; doi:10.1186/s40798-024-00722-8)
Supplement: Supplementary file 2 — Additional file 2. [file 40798_2024_722_MOESM2_ESM.docx]

**Supplementary File 1. Tools employed for the assessment of physical and psychosocial outcomes.**

*Instruments for assessing physical outcomes.*

1. Motor performance was primarily evaluated using the Test of Gross Motor Development-2, Test of Motor Proficiency second Edition (BOT-2), German Motor Test, Gross Motor Function Measure-88 and the Test of Gross Motor Development (TGMD).
2. Physical fitness was evaluated using a diverse range of instruments. Cardiorespiratory fitness (e.g., aerobic capacity) was assessed using the Bruce treadmill protocol or different progressive ergometer exercise tests, the 20-meter progressive aerobic cardiovascular endurance run (PACER), the six-minute walk test, or the Yo-Yo Intermittent Endurance test. Flexibility was evaluated using the sit and reach or Modified Thomas tests. Muscular strength in the upper body was evaluated using metrics such as the count of push-ups or curl-ups, hand-held dynamometers, and medicine-ball launches. Concurrently, lower-limb muscle strength was assessed through various means, including jumping tests such as the standing broad jump or vertical jump, as well as activities like lateral step-ups and sprint tests. Running speed and agility were gauged using the Shuttle run (10x5m) and the 25-ft walk/run test, and the speed of limb movement was measured using the Plate tapping test. Coordination was evaluated with the Agility t-test and the Timed Up and Go test. Explosive power was assessed through the Muscle Power Sprint Test (MPST), but also in jumping (Standing Broad Jump, Vertical Jump, counter movement jump) and throwing (Seated Throw) activities.
3. Finally, athletic competence was assessed through various skill tasks, such as dribbling and shooting tests, locomotor and ball skills, or the YMCA Water Skills Checklist.

*Instruments for assessing psychosocial outcomes.*

The instruments used were diverse, including the Child Behavior Checklist (CBCL), a widely used assessment tool measures behavioural and emotional problems in CaA. The CBCL assesses a wide range of behavioural and emotional domains, including internalizing problems (such as anxiety and depression), and social problems, and it also gathers information about adaptive functioning, such as social skills and activities. The School Social Behavior Scales (SSBS–2) or the Social Skills Improvement System (SSIS) were also used to evaluate these social skills. The Strengths-and-Difficulties-Questionnaire (SDQ) to assess a child’s emotional and behavioural well-being or the Goldstein Model to identify emotional regulation and interpersonal relationships were also used. The Social Responsiveness Scale (SRS-2) was one of the most common instruments used to measure social responsiveness and social-communication skills in individuals, and the Repetitive Behavior Scale-Revised (RBS-R) to measure the presence and severity of repetitive behaviours in individuals. Similarly, the Vineland Adaptive Behaviour Scales (VABS-2) measures adaptive behaviours in four main domains: Communication, Daily Living Skills, Socialization, and Motor Skills.

Other psychological included factors such as self-image concept and personality or perceived competence were assessed using the Self-efficacy Scale, Goal Attainment Scaling (GAS), Perceived Movement Skill Competence (PMSC), Children and Youth Physical Self-Perception Profile (CY-PSPP), The Children’s Self-Perceptions of Adequacy in and Predilection for Physical Activity (CSAPPA), self-esteem scale of Rosenberg or the Self-Perception Profile for Children (SPPC) that was used in different studies (66,69,74). The Physical Self-Inventory (PSI) was also used to assess various aspects of an individual’s physical self-concept. It is designed to evaluate one’s perceptions and attitudes towards physical appearance, abilities, and overall physical well-being. The Draw Self-Person List or the Body Shape Questionnaire (BSQ) were also used to assess the individual’s self-perception.

Another group of instruments used to measure various aspects of personality, behaviour, and psychological well-being included the IPAT 16 Personality Factors and the Eysenck Personality Inventory (EPI), which measures individual personality traits. The Test of Everyday Attention for Children (Tea-Ch) to identify attentional difficulties and the Behavior Assessment System for Children” (BASC) measures various domains, including emotional symptoms, adaptive skills, social skills, problem behaviours, and self-perceptions. The Mooney Problem Checklist is used to identify behavioral and emotional problems in CaA, and the Minnesota Multiphasic Personality Inventory (MMPI) is also used to assess various aspects of personality, including depression, anxiety, social introversion, and personality disorders.

Specific instruments such as the ADHD rating scale (ADHD-RSIV) and the German version of the Conners-3 were used to assess various domains of ADHD symptoms, such as inattention, hyperactivity, impulsivity, and peer relations. The Autism Rating Scale-Third Edition (GARS-3) scale to evaluate and measure the severity of ASD symptoms in individuals (e.g., social interaction, communication, and repetitive behaviors or restricted interests), together with the Chinese version of the Social Communication Questionnaire (SCQ) designed to gather information about a child's social communication skills and behaviors that are associated with ASD. In participants with obesity, the Salience of Weight-Related Issues Scale (SWRIS) was used to measure weight-related issues’ influence on an individual's self-perception and overall well-being.

Another group of variables focused on Executive Functioning and Attention Abilities. Multiple domains of attention, including selective attention, sustained attention, attentional control, and divided attention, were assessed with the Test of Everyday Attention for Children (Tea-Ch). To delve deeper into executive functions, various tests were carried out, among which the following stood out: Wisconsin Card Sorting Test (WCST), that assesses cognitive flexibility, set-shifting, and problem-solving abilities; The Stroop test, that measures cognitive flexibility and inhibition. The Childhood Executive Functioning Inventory (CHEXI) and the Delis-Kaplan Executive Function System (D-KEFS) focused on working memory, inhibition, cognitive flexibility, planning, and organization. Another specific test used to evaluate executive functions included the modified Simon Task, the Go/No-Go (GNG) task (inhibition), the modified Flanker task (switching), and the modified version of the color span backward task (updating). Finally, various studies used the digit span (forwards/backwards) and the letter–number-sequencing task of the HAWIK – IV to assess working memory. The Corsi block tapping task (CBTT) was also used to measure spatial working memory.

The last group focused on Health-Related Quality of Life (HRQoL). Both generic and disease-specific assessment tools were used in the included studies. The generic instrument more widely used was the Pediatric Quality of Life Inventory (PedsQL) (63,65,73,74,76). Other general instruments included the General health questionnaire (GHQ) (40), the Child Health Questionnaire (CHQ) (59), Pediatric Outcome Data Collection Instrument (PODCI) (43), KIDSCREEN-27-total score (33) or the Vécu et Santé Perçue de l’Adolescent et de l’enfant (VSP-A) (61). The disease-specific instruments (n = 4) included examples in asthma: Paediatric Asthma Quality of Life Questionnaire (PAQLQ) (64) or Cerebral Palsy (CP QOL-Child) (38). The Neuro-QoL pediatric assessments (44) were designed to assess HRQoL in CaA with neurological conditions.

| **TEST** | **OUTCOMES** | **DESCRIPTION** | **REFERENCES** |
| --- | --- | --- | --- |
| 10x 5 Meter Sprint Test | Running speed and agility | A test of speed and agility that involves sprinting 10 meters back and forth five times, with a 180-degree turn at each end | Clutterbuck et al. (2020) |
| A progressive treadmill exercise test | Cardiorespiratory fitness | A progressive treadmill exercise test is a cardiovascular fitness assessment that involves the participant walking or running on a treadmill at gradually increasing intensities. The goal is to measure the individual's endurance, aerobic capacity, and cardiovascular fitness. | Howell et al. (2018) |
| Back-saver sit-and-reach tests | Flexibility | The Back-Saver Sit-and-Reach test is a flexibility assessment that focuses on the flexibility of the lower back and hamstrings. The participant sits on the floor with legs fully extended and reaches forward toward their toes. The measurement is taken based on how far the participant can reach beyond their toes. | Pan et al. (2017) |
| Behavior Checklist for Children/Adolescents (CBCL) | Biopsychosocial profile | A questionnaire that assesses the behavioral and emotional problems of children and adolescents aged 6 to 18 years, based on parent or teacher ratings | Feitosa et al. (2017) |
| Bruce treadmill protocol | Aerobic capacity | A graded exercise test that involves increasing the speed and incline of a treadmill every three minutes until exhaustion, used to assess aerobic capacity and cardiovascular fitness | Verret et al. (2010) |
| Cerebral Palsy Quality of Life- Child (CP QOL-Child) | HRQoL | A questionnaire that measures the quality of life of children and adolescents with cerebral palsy aged 4 to 18 years, covering domains such as social well-being, participation, communication, health, access to services, pain, and family health | Clutterbuck et al. (2020) |
| Child Behavior Checklist (CBCL) | Behavioral problems | A widely used questionnaire that assesses the behavioral and emotional problems of children and adolescents aged 1.5 to 18 years, based on parent or teacher ratings | Benzing & Schmidt (2019), López-Diaz et al. (2021), Pan et al. (2010), Pan et al. (2017), Zoccolillo et al. (2015) |
| Child Health Questionnaire (CHQ) | HRQoL | A questionnaire that measures the physical and psychosocial well-being of children and adolescents aged 5 to 18 years, covering domains such as physical functioning, role/social limitations, emotional and behavioral problems, self-esteem, and family impact | Speyer et al. (2010) |
| Childhood Executive Functioning Inventory (CHEXI) | Executive function | The Childhood Executive Functioning Inventory (CHEXI) is a tool designed to assess executive functioning in children. The CHEXI specifically focuses on executive functioning in daily life and academic contexts | Wang et al. (2020) |
| Computer version of the Stroop Test | Executive function | Digital adaptation of the classic Stroop Test, a psychological test of selective attention and cognitive flexibility. In the Stroop Test, participants are presented with words written in different ink colors, and they are asked to name the ink color while inhibiting the automatic response to read the word itself. | Pan et al. (2017) |
| Coordination test | Coordination | A test of motor coordination that involves performing a series of tasks that require precise movements, such as tapping, tracing, or catching | Chiu et al. (2014) |
| Corsi block tapping task (CBTT) | Working memory | Neuropsychological test used to assess visuospatial short-term memory and spatial span. The task involves a series of blocks (usually nine) that are randomly arranged and presented to the participant. The blocks are often mounted on a board. | Yeung Any Tse et al. (2019) |
| Curl-up | Muscular endurance | A test of abdominal muscular endurance | Pan et al. (2017) |
| Curl-up /isometric push-up tests | Muscle endurance | Tests of muscular endurance | Fragala-Pinkham et al. (2011) |
| Delis-Kaplan Executive Function System (D-KEFS) | Executive function | A comprehensive set of tests for assessing executive functions | Howell et al. (2018) |
| Functional Mobility Scale | Mobility | A six-level scale that measures the functional mobility of children and adolescents with cerebral palsy, based on their ability to walk 5, 50, and 500 meters, with or without assistive devices | Clutterbuck et al. (2020) |
| General health questionnaire (GHQ) | HRQoL | A screening tool that detects psychological distress and mental health problems in individuals aged 16 to 65 years, covering domains such as somatic symptoms, anxiety, social dysfunction, and depression | Pourazar et al. (2018) |
| GMFM-Challenge | Gross motor skills | A set of 32 challenging items that complement the Gross Motor Function Measure-88 (GMFM-88) and assess the gross motor function of children and adolescents with cerebral palsy who have high levels of motor ability | Clutterbuck et al. (2022) |
| The Go/No-Go (GNG) task | Response inhibition | Used to measure inhibition and impulsivity. It is a type of continuous performance task where participants are required to respond selectively to certain stimuli while inhibiting responses to others. | Yeung Any Tse et al. (2019) |
| Golf skill assessments | Golf skills | Tools for assessing golf skills | Edwards et al. (2017) |
| Golf swing and golf putt | Golf skills | Specific skills in golf | Edwards et al. (2017) |
| Gross Motor Function Classification System | Mobility | A five-level classification system that describes the gross motor function of children and adolescents with cerebral palsy, based on their self-initiated movement, limitations, and need for assistive devices | Clutterbuck et al. (2020), Ross et al. (2017) |
| Gross Motor Function Measure-88 (GMFM-88) | Gross motor skills | A clinical tool that evaluates the gross motor function of children and adolescents with cerebral palsy or other neurological impairments, covering lying and rolling, sitting, crawling and kneeling, standing, and walking, running and jumping | Gercek et al. (2021) |
| HAAR checklist | Aquatic skill measures | A tool for assessing aquatic skills | Pan et al. (2010) |
| Impact on Family Scale | HRQoL | A questionnaire that measures the impact of a child’s chronic condition on the family’s functioning and well-being, covering domains such as financial burden, family cohesion, social isolation, and personal strain | Polat et al. (2020) |
| IPAT 16 Personality Factors | Personality | designed to measure and assess an individual's personality based on 16 primary personality traits. | Jette et al. (1977) |
| Isometric push-up | Muscular endurance | A test of upper body muscular endurance | Pan et al. (2017) |
| Jebsen– Taylor Test of Hand Function | Hand function | A test of hand function that involves performing seven simulated activities of daily living, such as writing, turning cards, feeding, stacking cans, picking up small objects, picking up large objects, and picking up heavy objects | Chiu et al. (2014) |
| Lateral step-up test | Muscular endurance | A test of lower limb strength and endurance that involves stepping up and down a platform or bench as many times as possible in 30 seconds | Gercek et al. (2021) |
| Long form of the Bruininks-Oseretsky Test of Motor Proficiency, Second Edition (BOT-2) | Motor skills | A comprehensive test that evaluates the motor skills of children and adolescents aged 4 to 21 years, such as fine manual control, manual coordination, body coordination, and strength and agility | Benzing & Schmidt (2019), Hakim et al. (2022), Lee et al. (2020), Pan et al. (2017), Rafiei Milajerdi et al. (2021), Wang et al. (2020) |
| Maximum voluntary isometric contraction | Muscle strength | A measure of muscle strength that involves exerting the maximum force possible against a fixed resistance, such as a dynamometer or a cable tensiometer | Chiu et al. (2014) |
| mCOPM activity goals | Physical competence | A modified version of the Canadian Occupational Performance Measure (COPM) that measures the performance and satisfaction of children and adolescents with cerebral palsy in relation to their self-identified activity goals | Clutterbuck et al. (2022) |
| Modified Ashworth Scale | Spasticity level | A clinical scale that measures the degree of spasticity in muscles, ranging from 0 (no increase in muscle tone) to 4 (affected part rigid in flexion or extension) | Gercek et al. (2021) |
| Movement Assessment Battery for Children-Second Edition (MABC-2) | Motor skills | A standardized test that assesses the motor performance of children and adolescents aged 3 to 16 years, covering manual dexterity, aiming and catching, and balance | Hamari et al. (2019), Rafiei Milajerdi et al. (2021), Ziereis & Jansen (2015) |
| Multidimensional Paediatric Evaluation of Disability Inventory Mobility scale (M-PEDI) | Mobility skills | a comprehensive assessment tool designed to evaluate the functional abilities and limitations of children with disabilities. The M-PEDI is specifically focused on assessing children's performance in daily activities and their level of independence. | Fragala-Pinkham et al. (2011) |
| Muscle Power Sprint Test (MPST) | Running speed and agility | A test of anaerobic performance and muscle power that involves sprinting 15 meters four times, with 10 seconds of recovery between each sprint | Clutterbuck et al. (2020) |
| Neuro-QoL pediatric assessments | HRQoL | A set of questionnaires that measure the health-related quality of life of children and adolescents with neurological conditions, covering domains such as fatigue, anxiety, depression, stigma, social participation, and physical function | Lai et al. (2022) |
| Ninehole Peg Test | Hand function | A test of fine motor skills and manual dexterity that involves picking up and placing nine pegs into nine holes on a board as quickly as possible, using one hand at a time | Chiu et al. (2014) |
| Pediatric Outcome Data Collection Instrument (PODCI) | HRQoL | A questionnaire that measures the functional status and quality of life of children and adolescents with musculoskeletal disorders, covering domains such as upper extremity function, transfers and mobility, sports and physical function, pain, and happiness | Feitosa et al. (2017) |
| Physical Self-Inventory (PSI-VSF) | Self-esteem | A questionnaire that measures the physical self-concept of children and adolescents aged 8 to 18 years, covering domains such as strength, endurance, flexibility, coordination, speed, and global physical self-worth | Saultier et al. (2021) |
| Programme satisfaction questionnaires | Satisfaction | Tools for assessing satisfaction with a specific program | Fragala-Pinkham et al. (2011) |
| Push-up test | Muscular endurance | A test of upper body muscular endurance that involves performing as many push-ups as possible in a given time or until fatigue | Cvetković et al. (2018), Gercek et al. (2021), Verret et al. (2010) |
| Quality of Upper Extremities Skills Test (QUEST) | Motor skills | A standardized test that measures the quality of movement of the upper extremities in children and adolescents with cerebral palsy, covering domains such as dissociated movements, grasp, protective extension, and weight bearing | Zoccolillo et al. (2015) |
| Repetitive Behavior Scale-Revised (RBS-R) | Repetitive behaviour | The primary purpose of the RBS-R is to provide a standardized and systematic way of assessing repetitive behaviors in individuals across different age groups. The scale covers a range of domains associated with repetitive behaviors, including stereotyped behavior, self-injurious behavior, compulsive behavior, ritualistic behavior, sameness behavior, and restricted behavior. | Wang et al. (2020) |
| School Social Behavior Scales (SSBS–2) | Social behaviour | A measure of social competence and antisocial behavior in children | Pan et al. (2010) |
| Seated Throw | Explosive power | A test of upper body explosive power that involves throwing a medicine ball as far as possible from a seated position, with both hands holding the ball at chest level | Clutterbuck et al. (2022) |
| Self-Perception Profile for Children (SPPC) | Self-perception | The SPPC typically assesses children between the ages of 8 and 14. It consists of separate subscales, each focusing on a specific dimension of self-perception. | Cliff et al. (2007), Edwards et al. (2017) |
| Simple Reaction Time (SRT) and Discriminative Reaction Time (DRT) | Cognitive function | Two tests of reaction time that measure the speed of responding to a stimulus, either with a single response (SRT) or with different responses depending on the stimulus (DRT) | Pourazar et al. (2018) |
| Sit and reach test | Flexibility | A common measure of flexibility that involves sitting on the floor with legs extended and reaching forward as far as possible with the hands | Cvetković et al. (2018), Feitosa et al. (2017), García-Gómez et al. (2013), Gercek et al. (2021), Pan et al. (2017), Verret et al. (2010) |
| Sit-up test | Muscular endurance | A test of abdominal muscular endurance that involves performing as many sit-ups as possible in a given time or until fatigue | Cvetković et al. (2018), Gercek et al. (2021), Verret et al. (2010) |
| Six-minute walk test | Aerobic capacity | A submaximal exercise test that measures the distance walked in six minutes, used to assess functional capacity and endurance | Gercek et al. (2021), Ross et al. (2017) |
| Six-minute walk test (6 MWT) | Cardiorespiratory fitness | A submaximal exercise test that measures the distance walked in six minutes, used to assess functional capacity and endurance | Saultier et al. (2021) |
| Social Communication Questionnaire (SCQ) | Social communication skills | is a screening tool designed to assess the presence of social communication difficulties and behaviors associated with autism spectrum disorder (ASD). | Lee et al. (2020) |
| Social Responsiveness Scale Second Edition (SRS-2) | Social responsiveness | A questionnaire that measures the severity of social communication difficulties and autism spectrum disorder symptoms in individuals aged 2.5 to 18 years, based on parent or teacher ratings | Benzing & Schmidt (2019), Guest et al. (2017), Morales et al. (2021), Pan et al. (2017), Wang et al. (2020), Yeung Any Tse et al. (2019) |
| Social Skills Improvement System (SSIS) | Social skills | A measure of social skills and problem behaviors in children | Guest et al. (2017) |
| Standing Broad Jump | Explosive power | A test of lower body explosive power that involves jumping as far as possible from a standing position, with feet together and arms swinging | Clutterbuck et al. (2022) |
| Static balance test | Balance | A test of postural stability that involves standing on one leg with eyes open or closed for as long as possible, without losing balance | Gercek et al. (2021) |
| Stroop Color and Word Test | Inhibition | A classic test of executive function that measures the ability to inhibit a dominant response and switch between tasks, by naming the color of words that are either congruent or incongruent with their meaning | Benzing & Schmidt (2019), Kadri et al. (2019) |
| Test of Everyday Attention for Children (Tea-Ch) | Attention functions | A standardized test that measures different aspects of attention, such as selective attention, sustained attention, and shifting attention, in children aged 6 to 16 years | Verret et al. (2010) |
| Test of Gross Motor Development (TGMD) | Motor skills | A standardized test that measures the gross motor skills of children aged 3 to 10 years, such as running, jumping, throwing, and catching | Bo et al. (2019), Cliff et al. (2007), Edwards et al. (2017), Griffin et al. (2013), Guest et al. (2017), Verret et al. (2010) |
| Test of Gross Motor Development-3 (TGMD-3) | Gross motor skills | designed to measure the gross motor skills of children aged 3 to 10 years. It is the third edition of the Test of Gross Motor Development series and is commonly employed in educational, clinical, and research settings. The TGMD-3 provides a systematic and comprehensive evaluation of a child's proficiency in fundamental motor skills, which are essential for everyday activities and physical development. | Lee et al. (2020) |
| Test of Gross Motor Development-second edition (TGMD-2) | Gross motor skills | A standardized test that measures the gross motor skills of children aged 3 to 10 years, such as running, jumping, throwing, and catching | Clutterbuck et al. (2020) |
| The 20-m progressive aerobic cardiovascular endurance run (PACER) | Cardiovascular fitness | A measure of aerobic capacity | Pan et al. (2017) |
| The Children’s Self-Perceptions of Adequacy in and Predilection for Physical Activity (CSAPPA) | Self-efficacy | A measure of children’s self-perceptions related to physical activity | Edwards et al. (2017) |
| The Pediatric Quality of Life Inventory version 4.0 (PedsQL) | HRQoL | A measure of health-related quality of life in children | Griffin et al. (2013), Lofrano-Prado et al. (2022) |
| The Pictorial Scale of Perceived Movement Skill Competence (PMSC) | Perceived competence | A measure of children’s perceived motor competence | Edwards et al. (2017) |
| The Self-Concept Test | Self-concept | A measure of self-concept | Jette et al. (1977) |
| The Vineland Adaptive Behaviour Scales, 2nd edition (VABS-2) | Adaptive behaviour | A measure of adaptive behavior in individuals with intellectual disability | Guest et al. (2017) |
| Timed Up and Go (TUG) | Mobility | A test of functional mobility and fall risk that involves rising from a chair, walking three meters, turning around, walking back, and sitting down again, as fast as possible | Clutterbuck et al. (2020) |
| Vécu et Santé Perçue de l’Adolescent et de l’enfant (VSP-A) | HRQoL | A questionnaire that measures the perceived health and quality of life of children and adolescents aged 8 to 18 years, covering domains such as physical well-being, psychological well-being, relations with parents, relations with friends, school environment, and global quality of life | Saultier et al. (2021) |
| Vertical Jump | Explosive power | A test of lower body explosive power that involves jumping as high as possible from a standing position, with one arm reaching up | Clutterbuck et al. (2022) |
| Visual Pain Analog Scale | Pain | A scale that measures the intensity of pain by asking the individual to mark a point on a line that ranges from no pain to worst pain imaginable | Polat et al. (2020) |
| Visual-Motor Integration functioning scale (VIM) | Visual-motor integration | A standardized test that measures the ability to coordinate visual and motor skills, such as copying shapes, letters, and numbers | Zoccolillo et al. (2015) |
| Wechsler Abbreviated Scale of Intelligence (WASI) | General intelligence | Intelligence test designed to provide a reliable and valid measure of cognitive abilities. | Howell et al. (2018) |
| Wisconsin Card Sorting Test (WCST) | Executive function | is a neuropsychological test designed to assess cognitive flexibility, executive function, and the ability to adapt to changing situations.The primary objective of the WCST is to measure the respondent's ability to form abstract concepts, shift cognitive strategies, and adapt to changing rules. | Pan et al. (2017), Wang et al. (2020) |
| YMCA Water Skills Checklist | Swimming skills | A checklist that measures the aquatic skills of children and adolescents, such as entering and exiting the water, floating | Fragala-Pinkham et al. (2011) |
